# Supplementary material for: Language-based EMA assessments help understand problematic alcohol consumption
Source: PLoS One. 2024 Mar 6;19(3):e0298300. doi: 10.1371/journal.pone.0298300 (PMC10917301; doi:10.1371/journal.pone.0298300)
Supplement: S1 Appendix — (DOCX) [file pone.0298300.s001.docx]

**Supporting information for**

**Language-based EMA Assessments Help Understand Problematic Alcohol Consumption**

**Table S1. Between-person correlations**

**
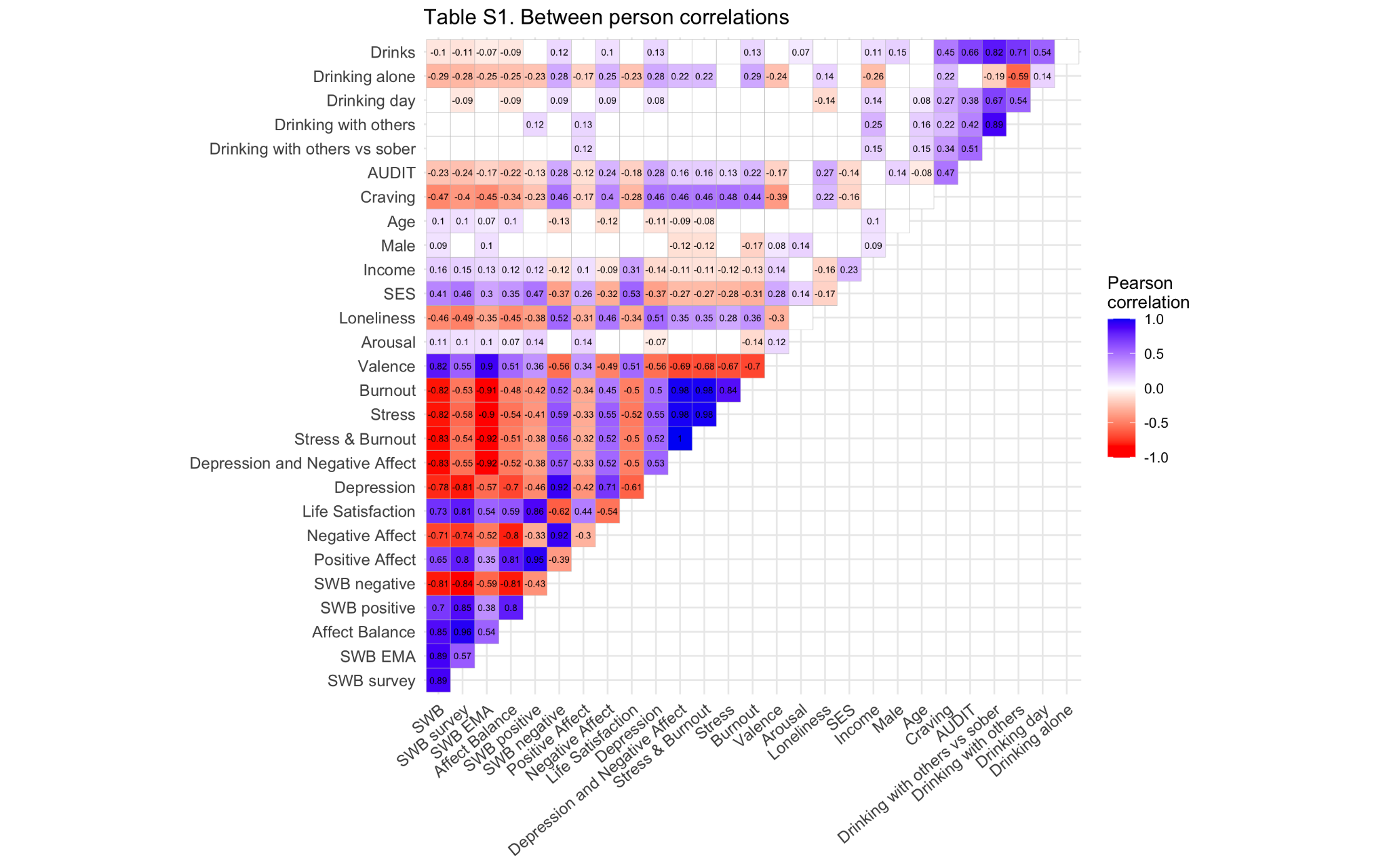
**

*Note:* SWB = Subjective Well-Being. Craving asks to indicate agreement from 0 to 10 on the item “I have a strong urge to drink”. SES = Socioeconomic status. Positive and Negative Affect are from the PANAS scales. White squares were non-significant. Drinks were Anscombe transformed. *N* = 187 - 908.

**Table S2. Within-person correlations for drinking tonight**

**
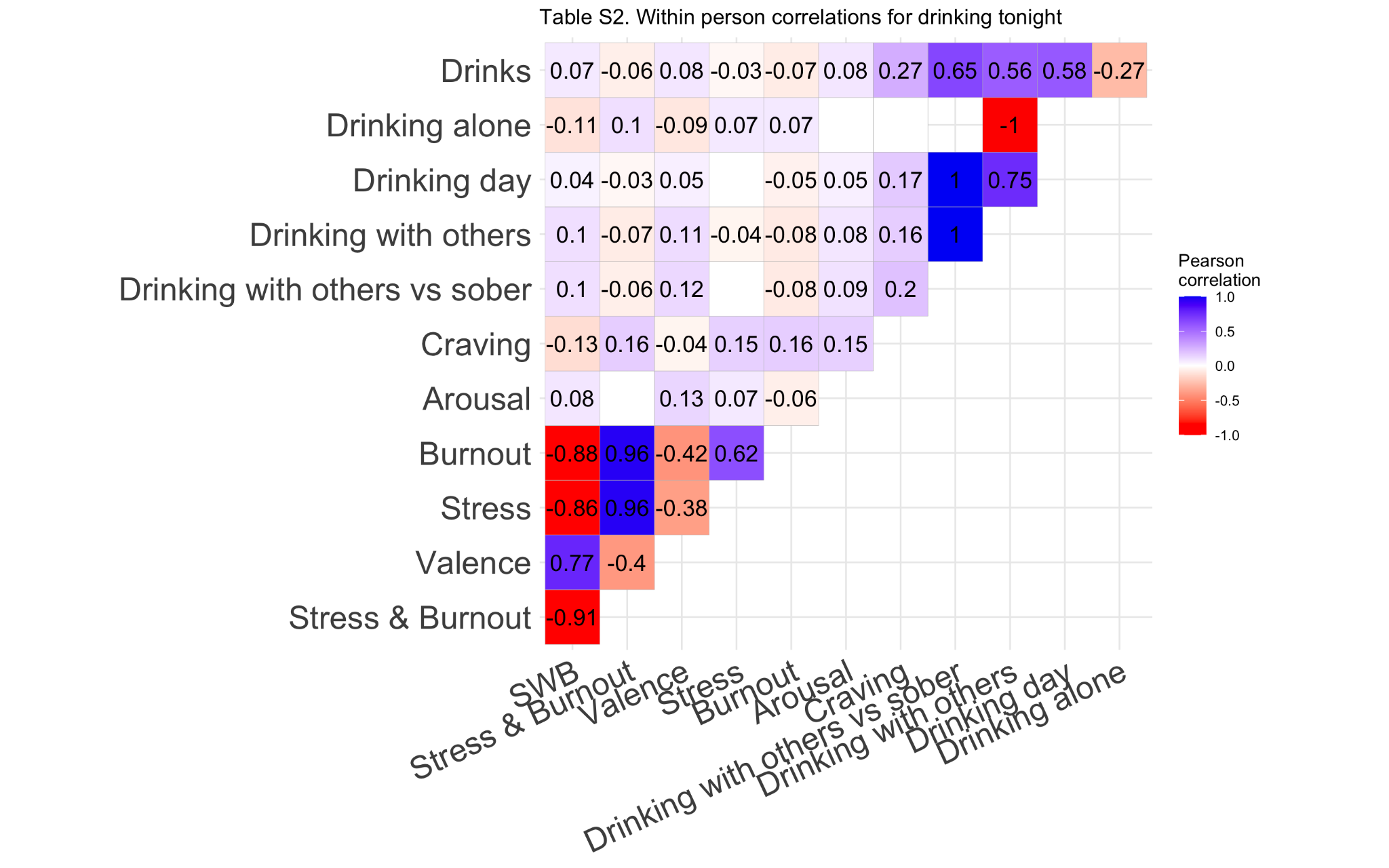
**

*Note:* SWB = Subjective Well-Being. Craving asks to indicate agreement from 0 to 10 on the item “I have a strong urge to drink”. Drinks were Anscombe transformed. *N* = 1436 - 10171.

**Table S3. Within-person correlations for drinking yesterday**

**
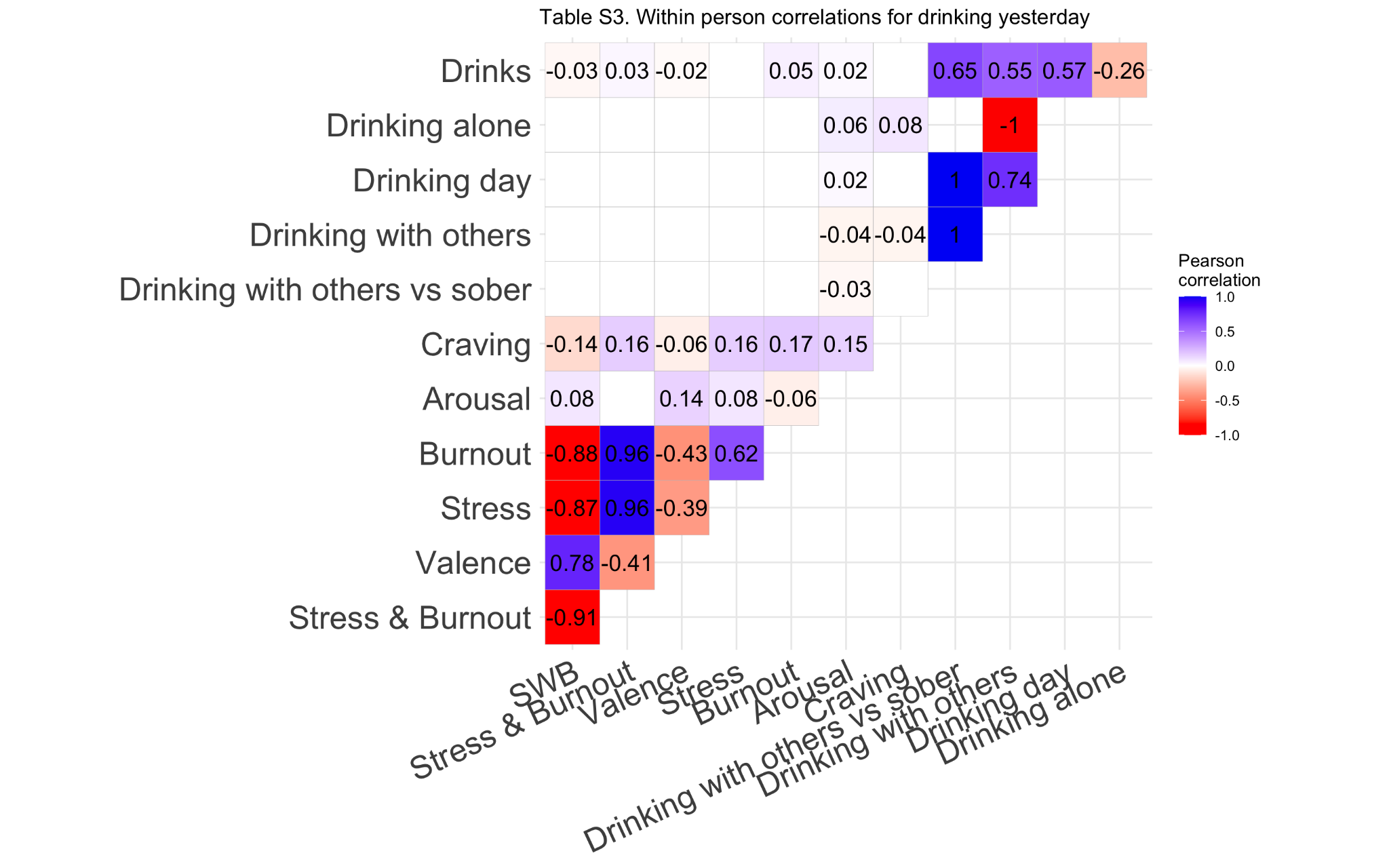
**

*Note:* SWB = Subjective Well-Being. Craving asks to indicate agreement from 0 to 10 on the item “I have a strong urge to drink”. Drinks were Anscombe transformed. *N* = 1713 - 12692

**Multilevel modeling joint mode**

We ran joint mode multilevel models of the same variables depicted in Table 1b-c, meaning that the models were fully specified (this is like running a bivariate linear regression, and the manuscript involved equivalent correlations). The multilevel fixed effects in the S4 Table contain standardized beta coefficients identical to the correlations depicted in Table 1b-c For the relationship between SWB and drinking alone and drinking day, we added SWB as the criterion because these drinking variables are binary. The odds ratios from the equivalent logistic regressions did not deviate from the results presented here.

**Table S4. Fixed effects from joint mode multilevel models.**

**
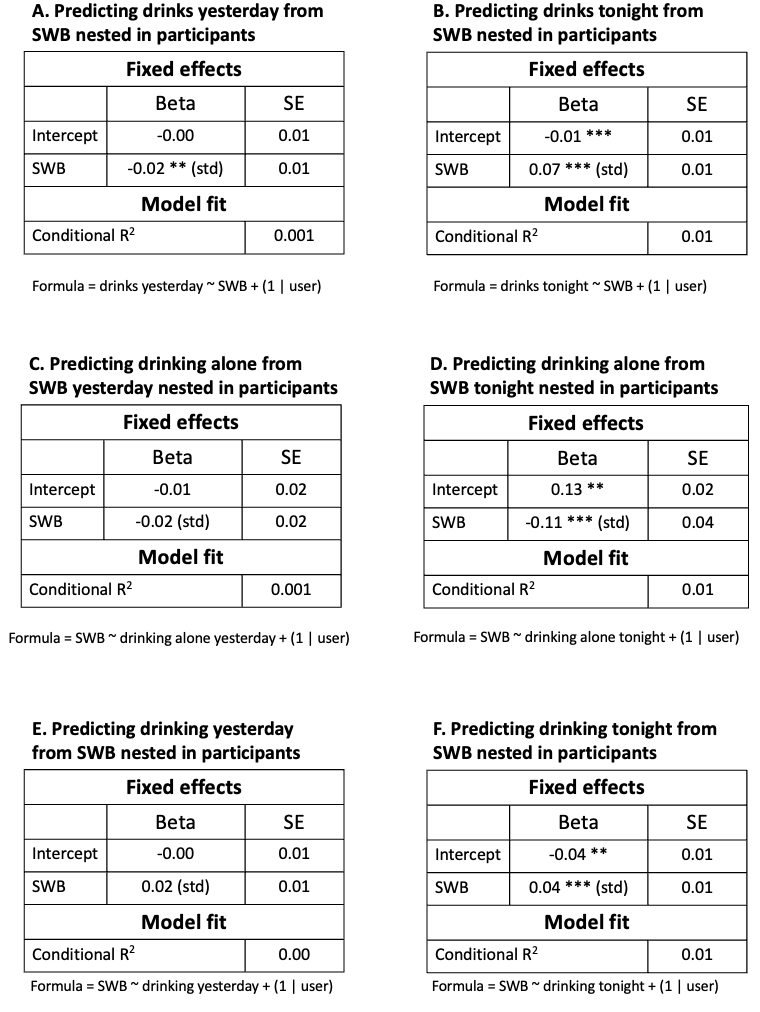
**

We also ran a series of more complex multilevel models. First, by predicting within-centered SWB from within-centered drinking and grand mean-centered AUDIT (S5a-b Table). The fixed effect of AUDIT helped predict SWB besides drinking behavior yesterday (5a) but not besides drinking behavior tonight (5b).

**Table S5. Multilevel models predicting within-centered well-being**

**
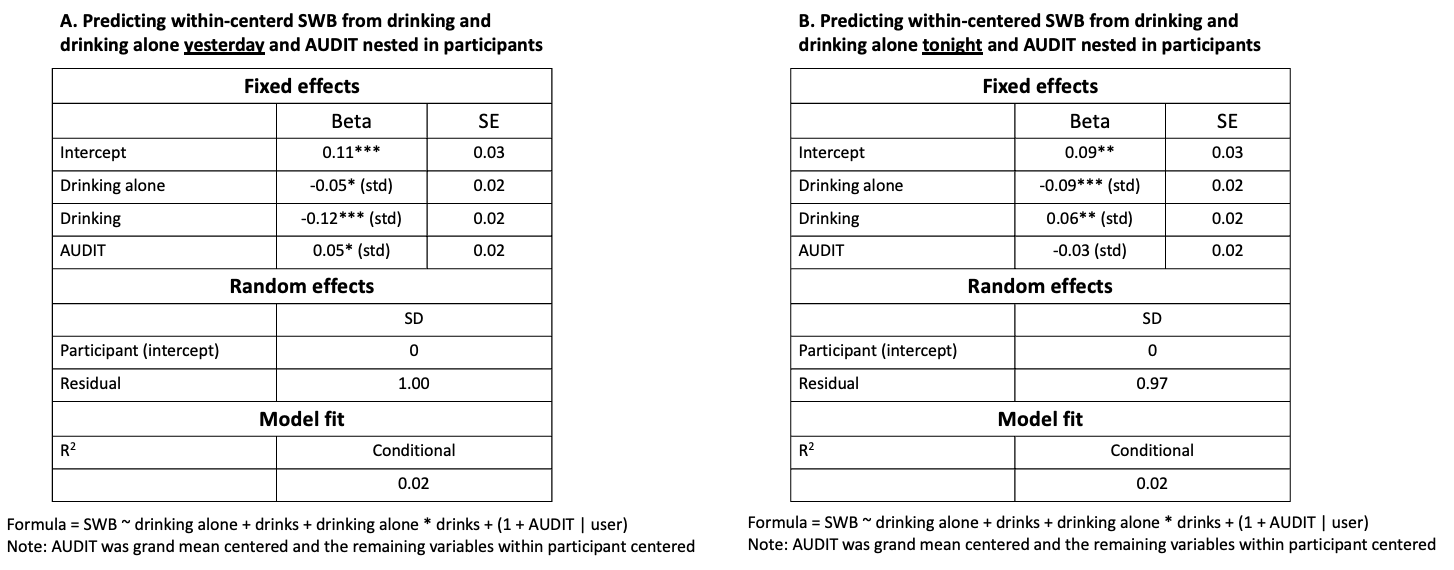
**

When grand mean centering SWB, drinking behaviors, and AUDIT, AUDIT was a strong predictor, especially besides drinking tonight variables (S6 Table). As in Table 1 and S4-5 Tables, drinking yesterday related to lower SWB but not drinking tonight, whereas drinking alone tonight related to lower SWB but not drinking alone yesterday.

**Table S6. Multilevel models predicting grand mean-centered well-being**

**
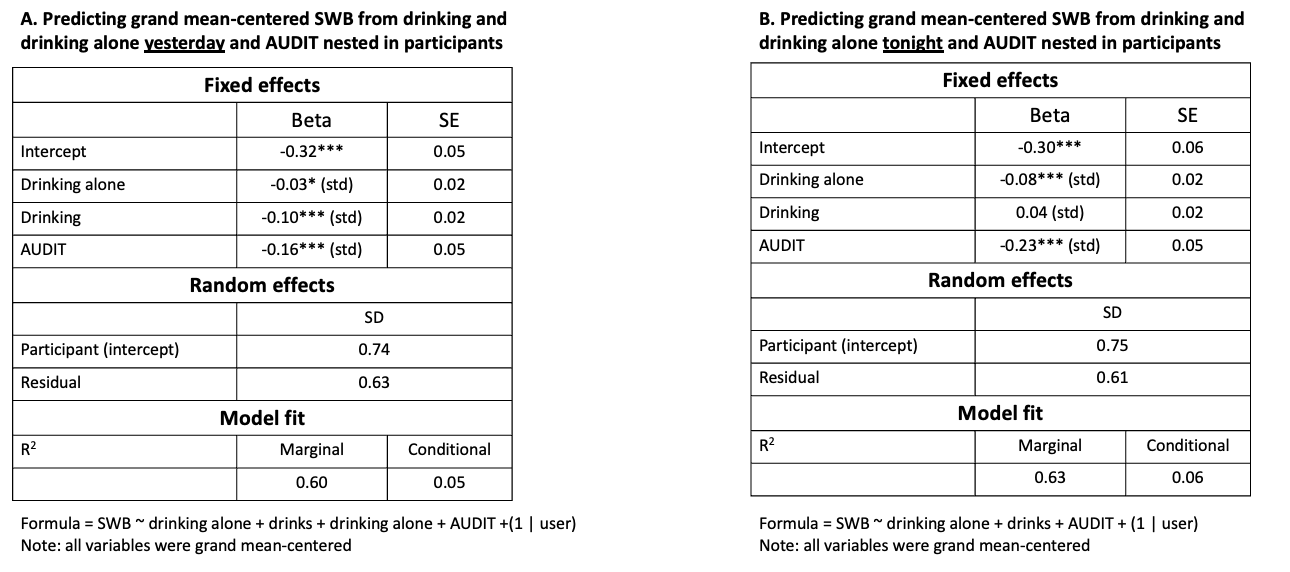
**

Finally, we tested whether there was an interaction between drinking and drinking alone and random effects of AUDIT (S7 Table). There was a significant interaction effect between drinking and drinking alone, indicating that drinking alone tonight relates to lower SWB, but this is moderated by the number of drinks. As expected, there was a strong random effect of AUDIT on SWB (*r* = -.47 and -.52). For all the joint mode multilevel models, we used the lme4 [1], broom.mixed [2], easystats [3], and lmerTest [4] packages.

**Table S7. Mixed multilevel models predicting grand mean-centered well-being
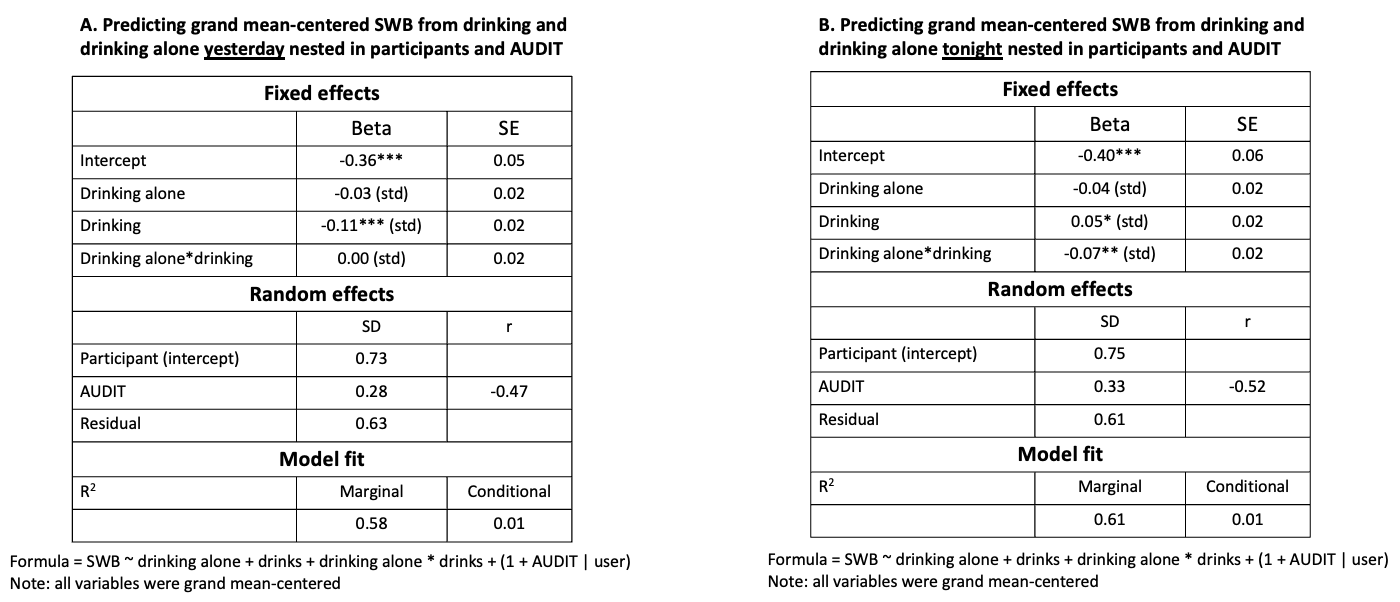
**

| **Table S8. Scatterplots of Subjective Well-Being and drinking** |
| --- |
| 1. **Raw mean drinking and Subjective Well-Being** |
| **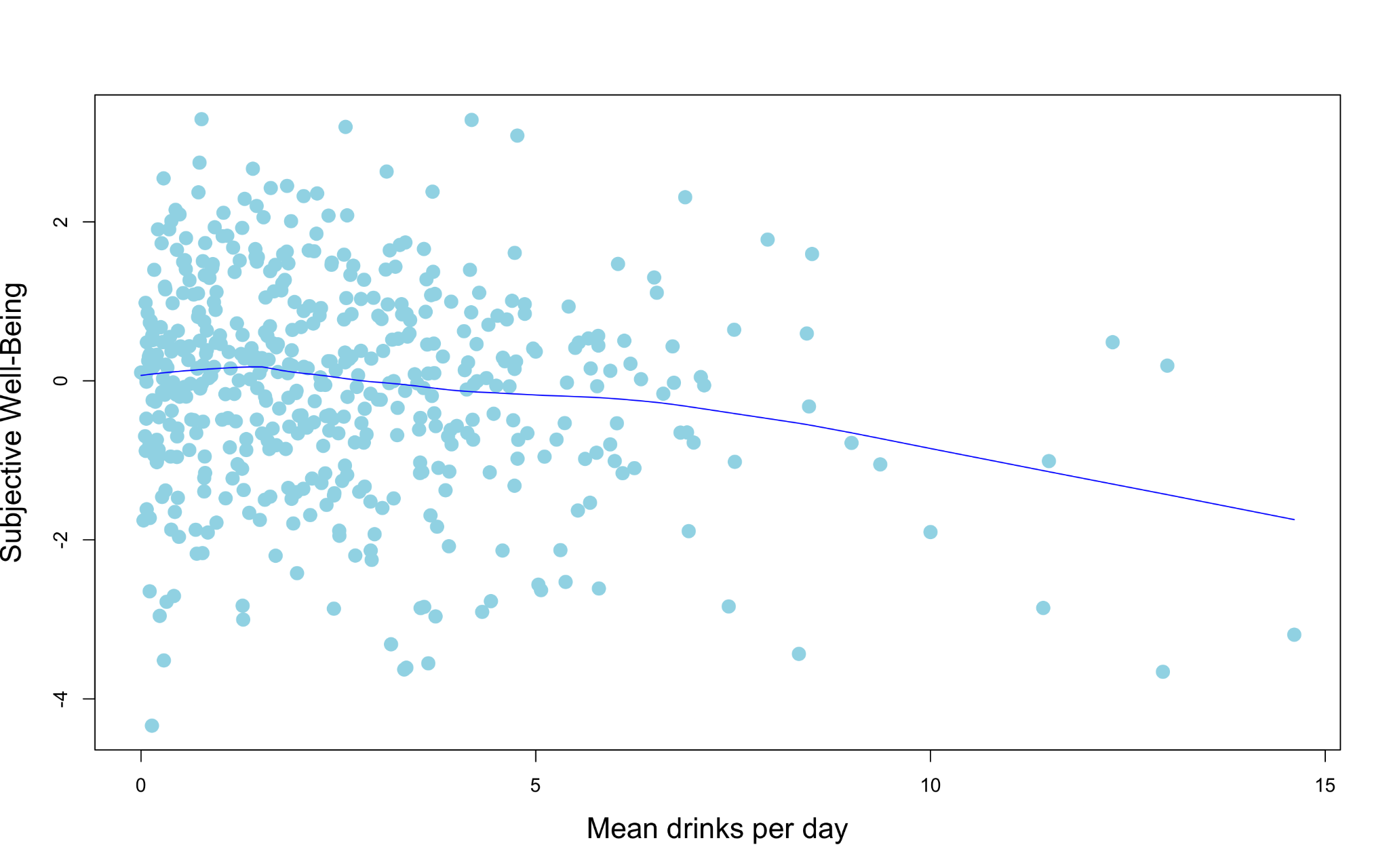** |
| *Note: n* = 494. Each dot represents the mean of one specific score. Thus, there was only one dot for sober people. |
| 1. **AUDIT score and Subjective Well-Being** |
| **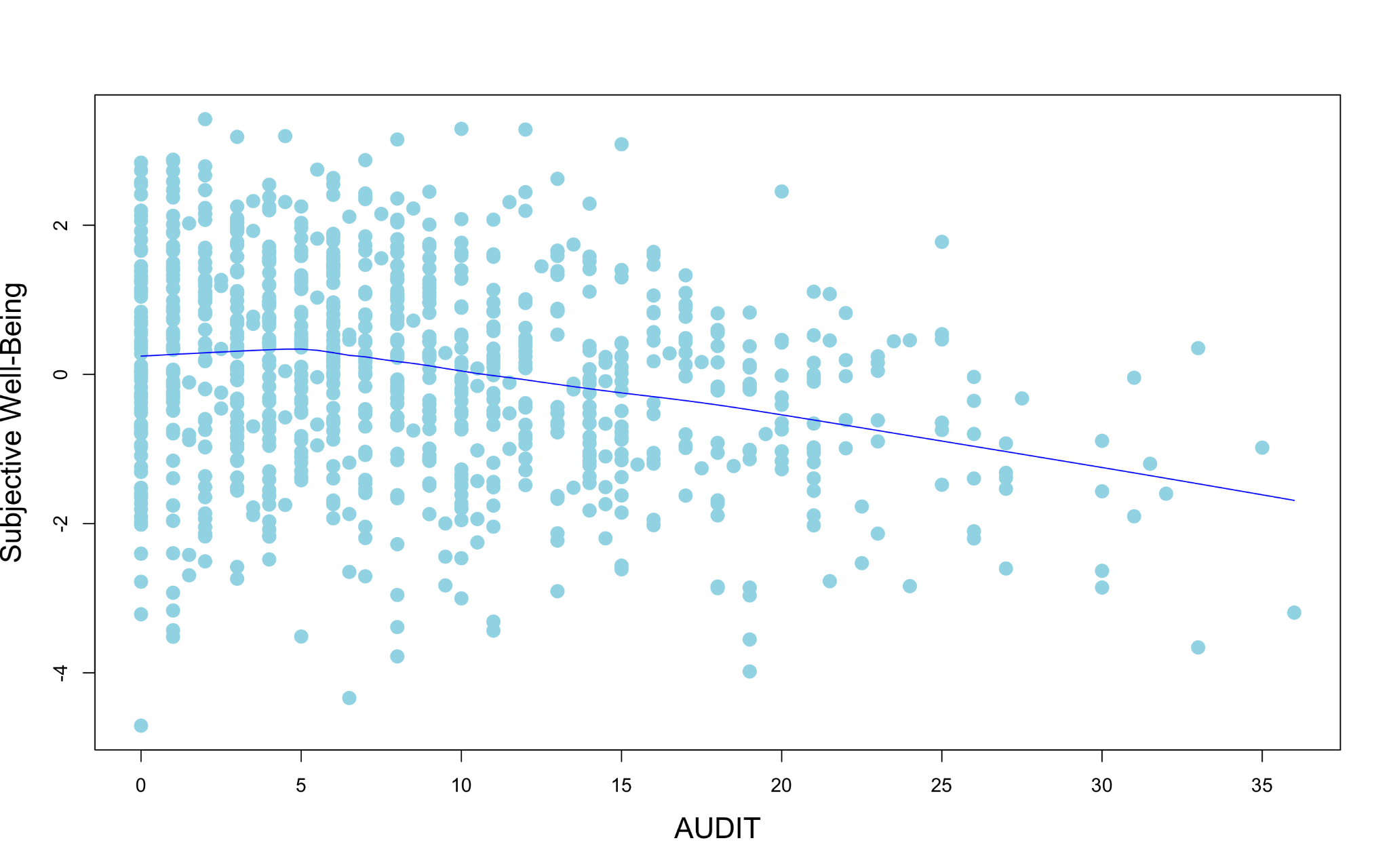** |
| *Note: N* = 891 |

**Table S9. Hierarchical Multiple Regression model for predicting Subjective Well-Being**

| **Predictors (β)** | | | | | |
| --- | --- | --- | --- | --- | --- |
| **Age and gender^1^** | **+Drinks^1^** | **+AUDIT^1*^** | **+Drinking alone^2**^** | **+Loneliness^3**^** | **adj R^2^ (*r*)** |
| .08 and .10 |  |  |  |  | .01 (.13) |
| .10 and .10 | -.12 |  |  |  | .03 (.18) |
| .07 and .12 | NS | -.29 |  |  | .07 (.28) |
| NS | NS | -.28 | -.30 |  | .14 (.40) |
| NS | NS | -.20 | -.28 | -.41 | .34 (.60) |

*Note: ^1^ = n* = 878, ^2^ = *n* = 221, ^3^ = *n* = 184. * = AUDIT was not perfectly linear in this model, and the model showed some homoscedasticity. ** = The already Anscombe transformed drinking variable was not perfectly linear in these models.

**Table S10. All language related to drinking and AUDIT**

**
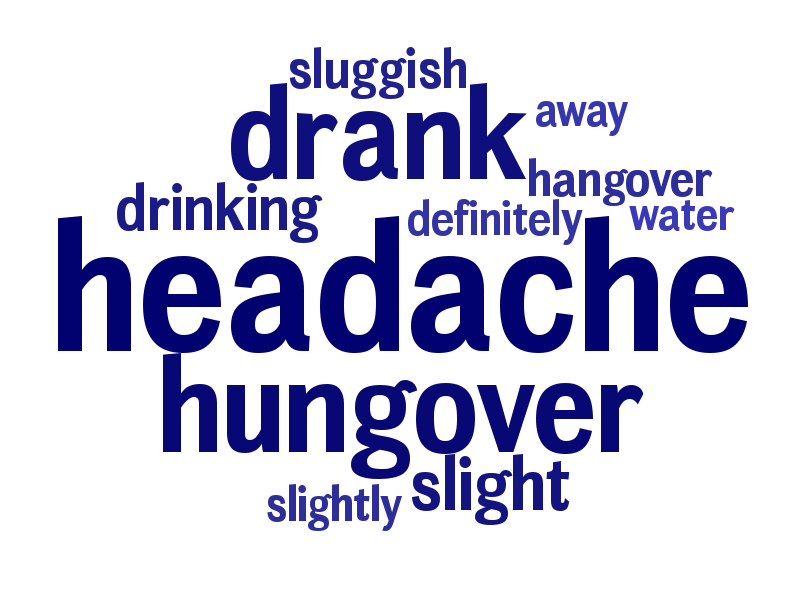

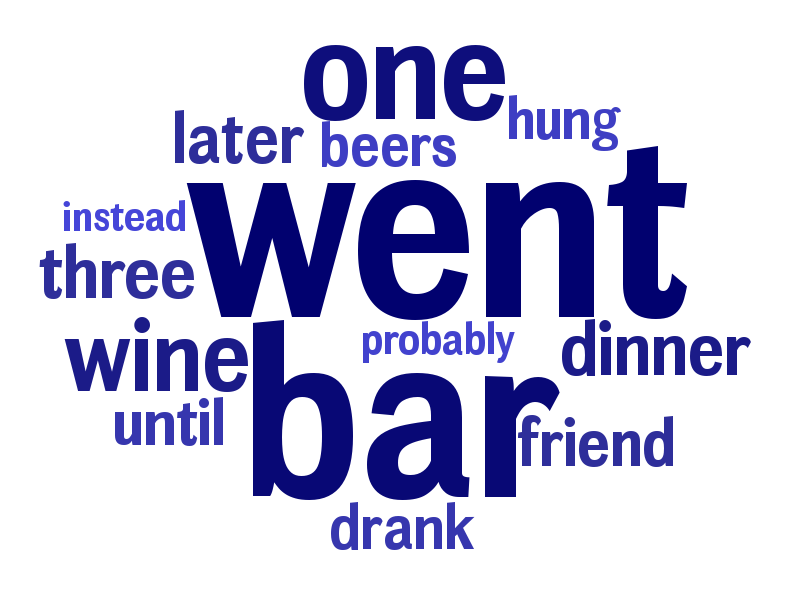

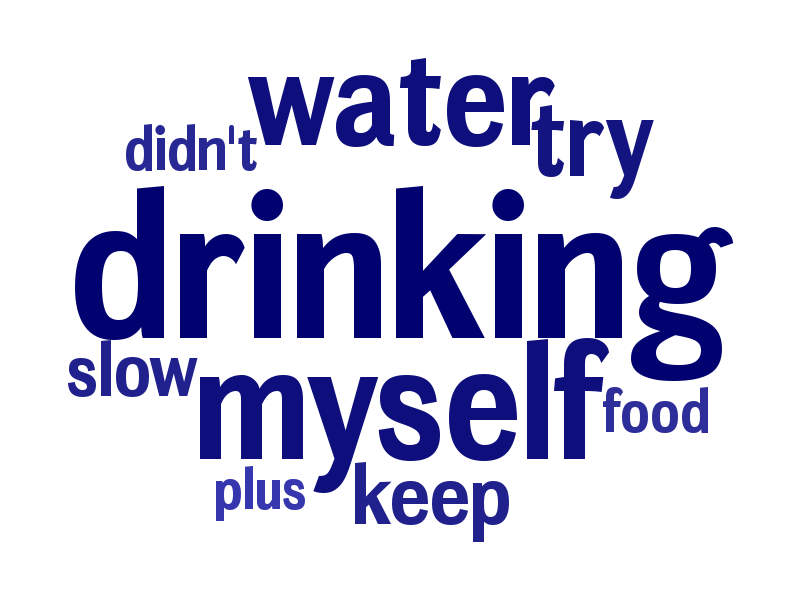

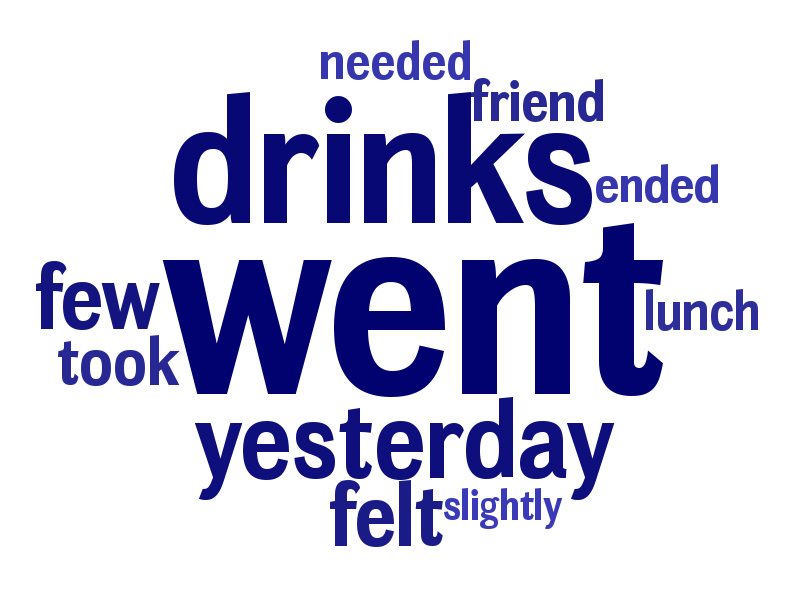
 Audit** *r* = .24 (.23) *r* = .21 (.21) *r* = .22 (.22) *r* = .20 (.19)

**Betw** *r* = .18 (.20) *r* = .25 (.24) *r* = .23 (.22) *r* = .25 (.24)

**W-yes** *r* = .13 *r* = .10 *r* = .13

**W-ton**  *r* = .05

**
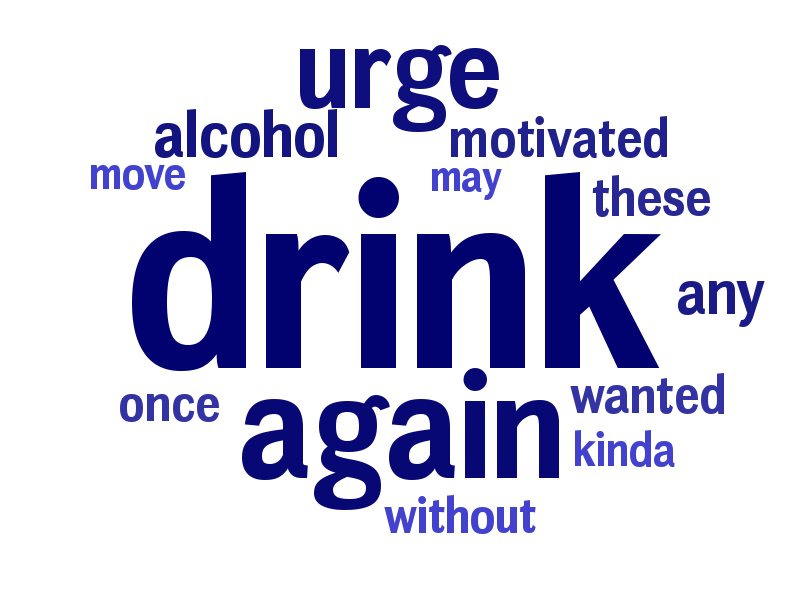

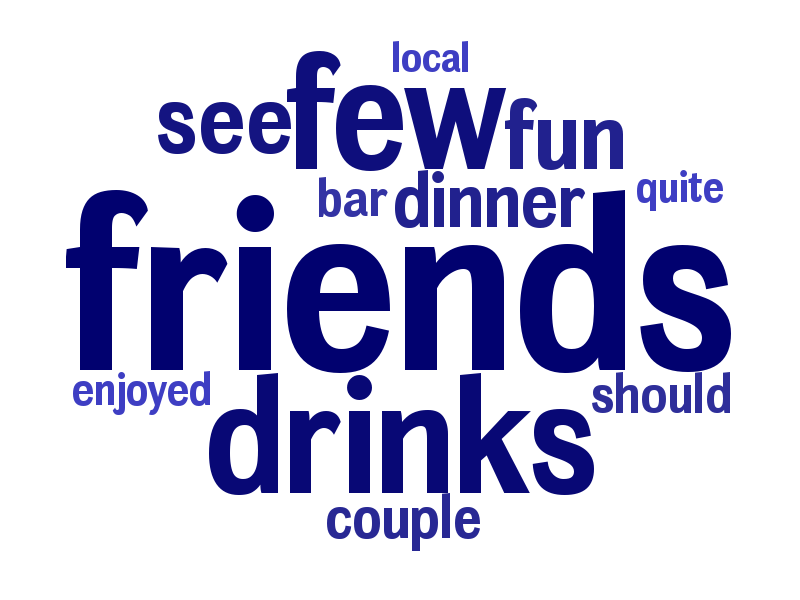

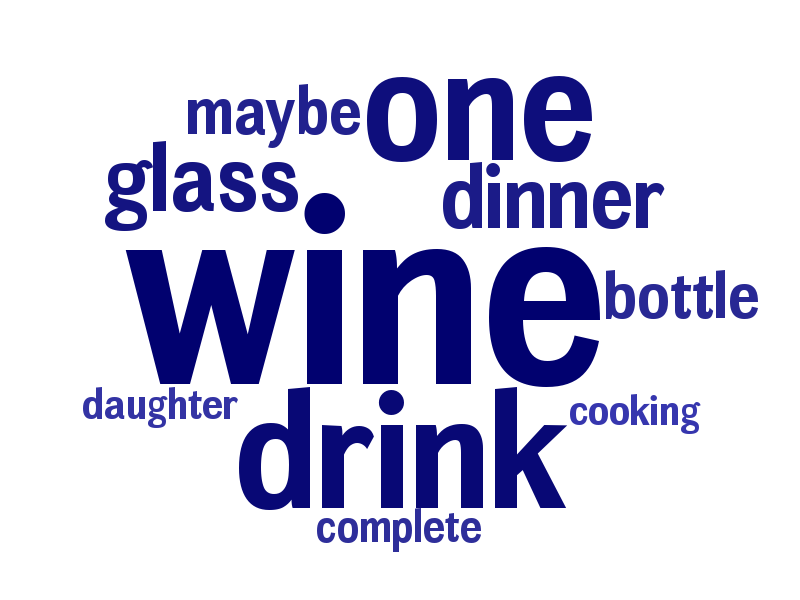

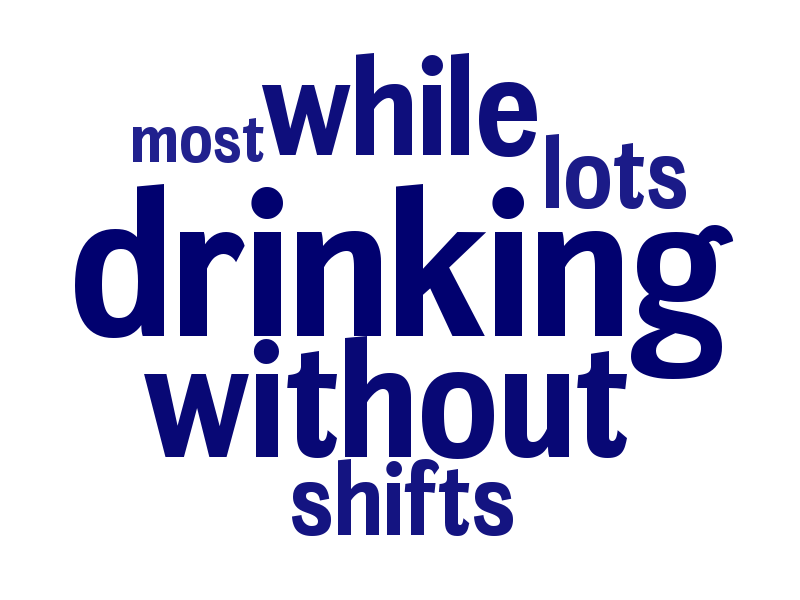
**

**AUDIT** *r* = .20 (.17) *r* = .15 (NS) *r* = .14 (.14) *r* = .19 (.18)

**Betw** *r* = .14 (NS) *r* = .26 (.24) *r* = .18 (.16) *r* = .17 (.15)

**W-yes**  *r* = .11

**W-ton** *r* = .07

**
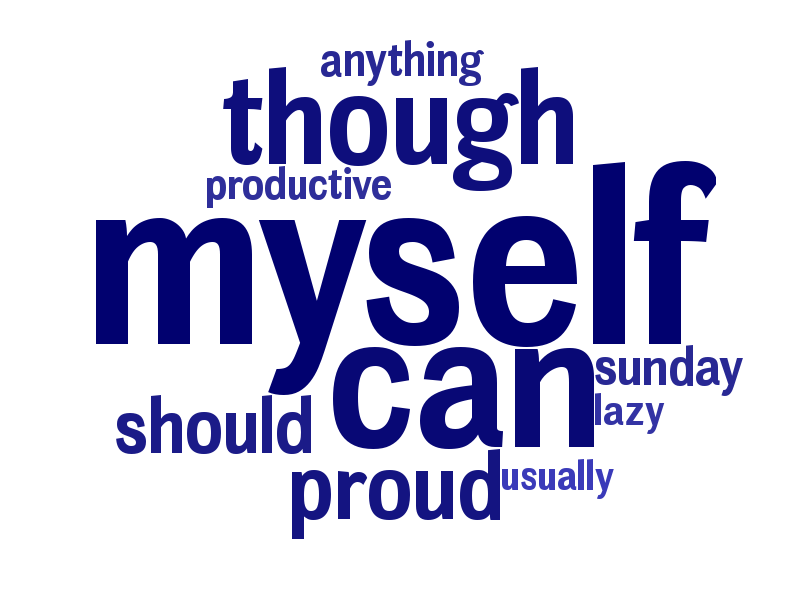

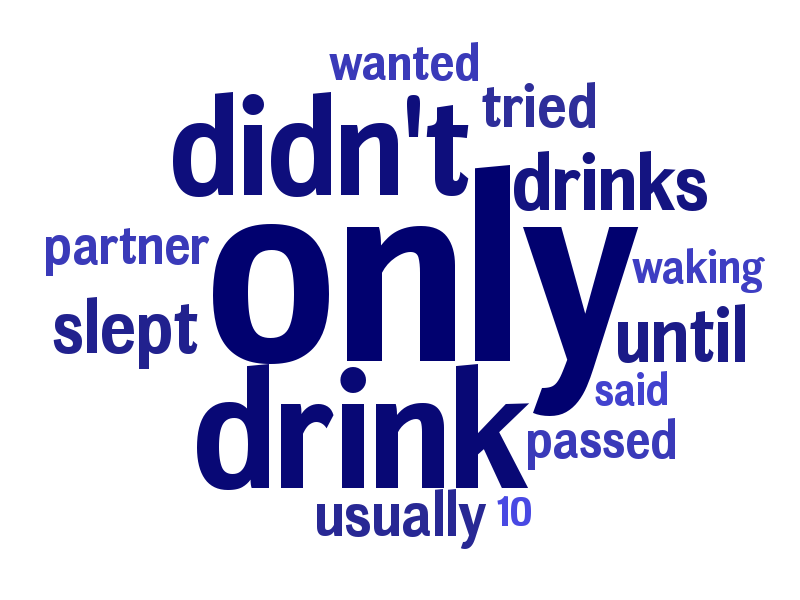

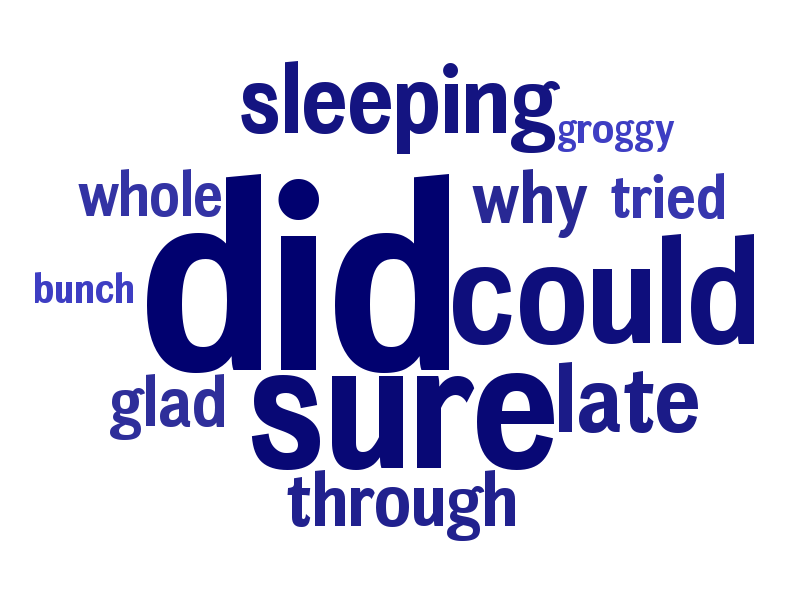

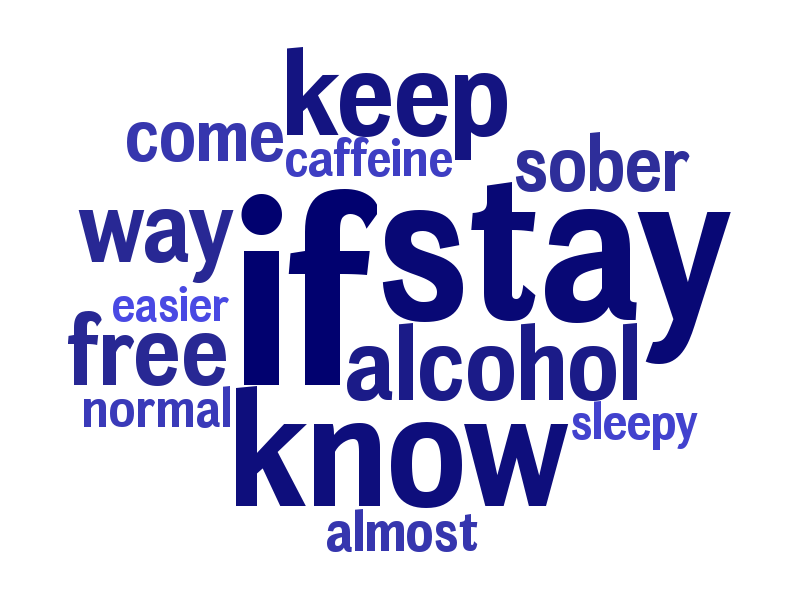

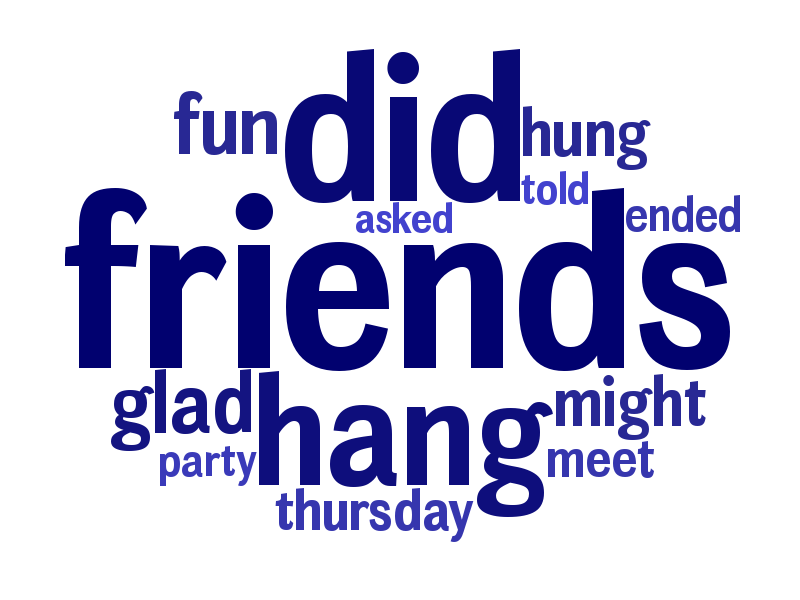
**

**AUDIT** *r* = .17 (.16) *r* = .13 (NS) *r* = .16 (.15) *r* = .16 (NS)

**Betw** *r* = .18 (.16) *r* = .18 (.17)

**W-yes** *r* = .10

**W-ton** *r* = .06

**
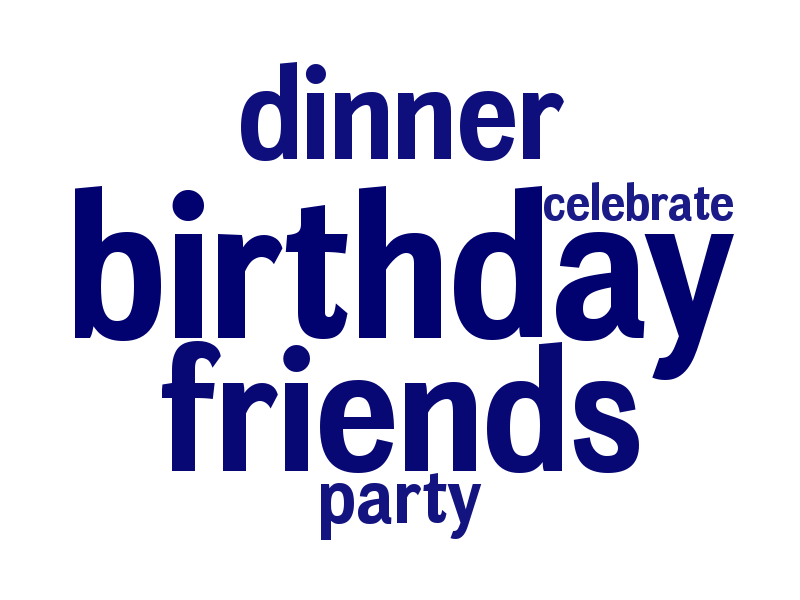

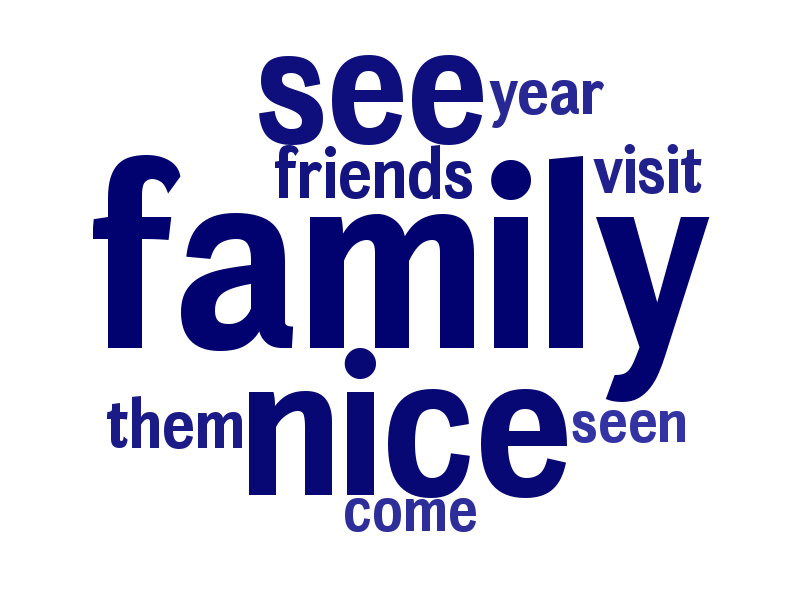
**

**W-yes** r = .10 r = .09

**W-ton** *r* = .08 r = .05

**
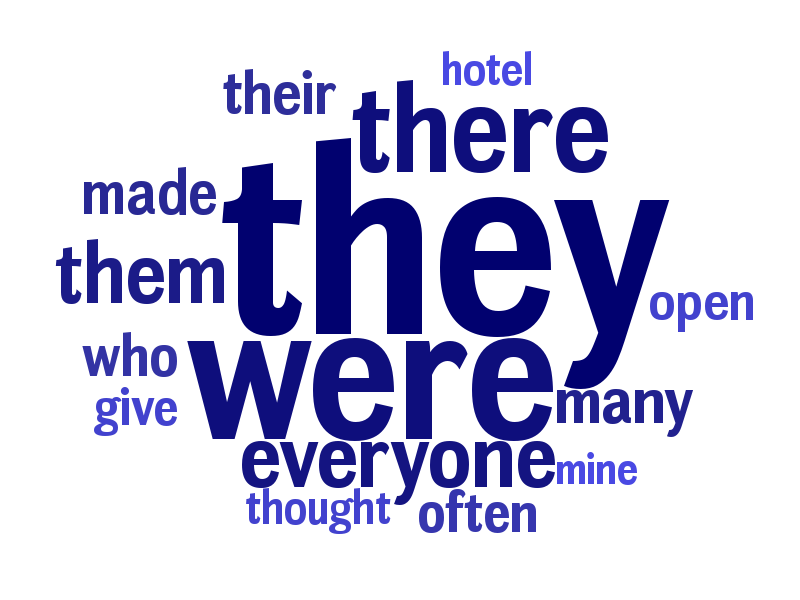

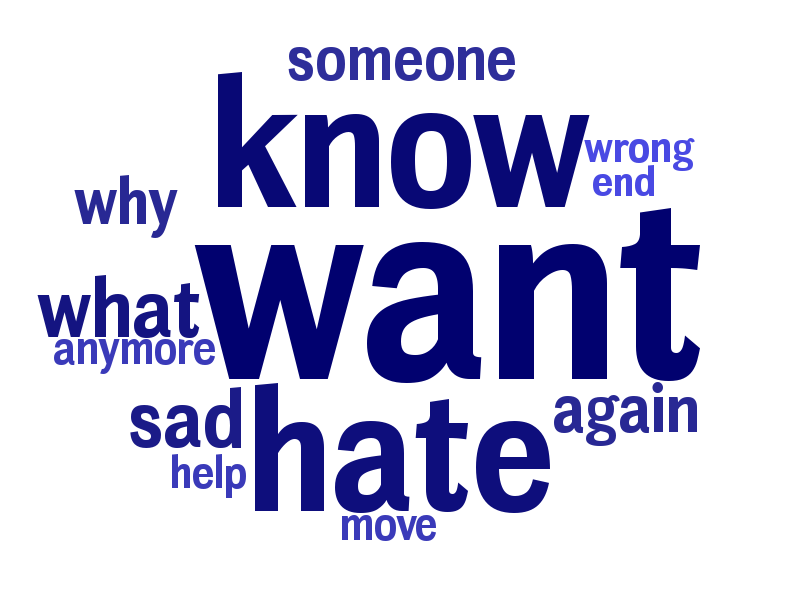

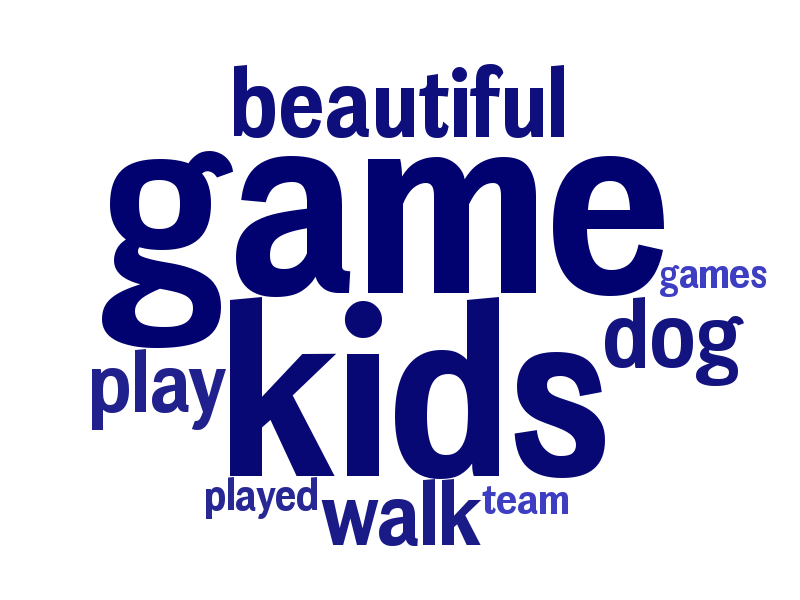

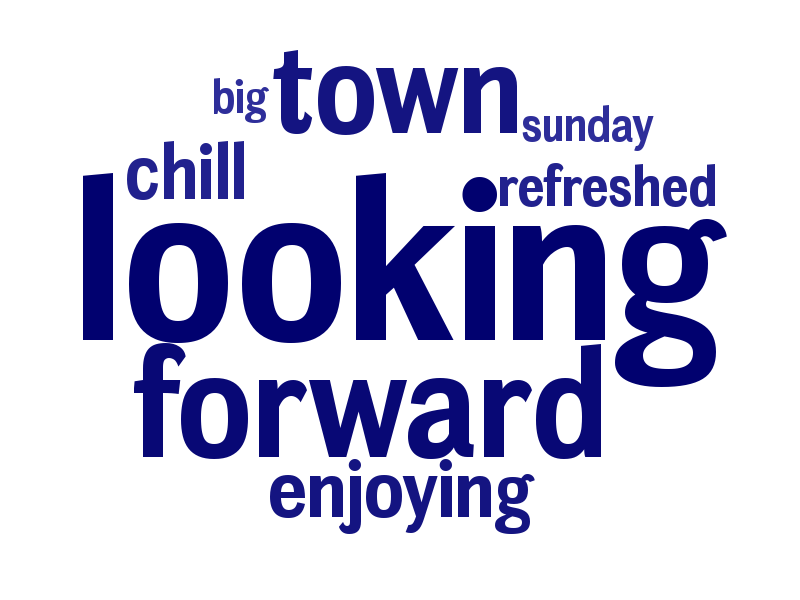
**

**W-yes**  r = .04 r = .05

**W-ton** *r* = .05 *r* = .05

*Note:* betw = between person analysis, W-yes = within person correlation to drinking yesterday, W-ton = within person correlation to drinking tonight.

**Table S11. All language related to drinking alone**

**
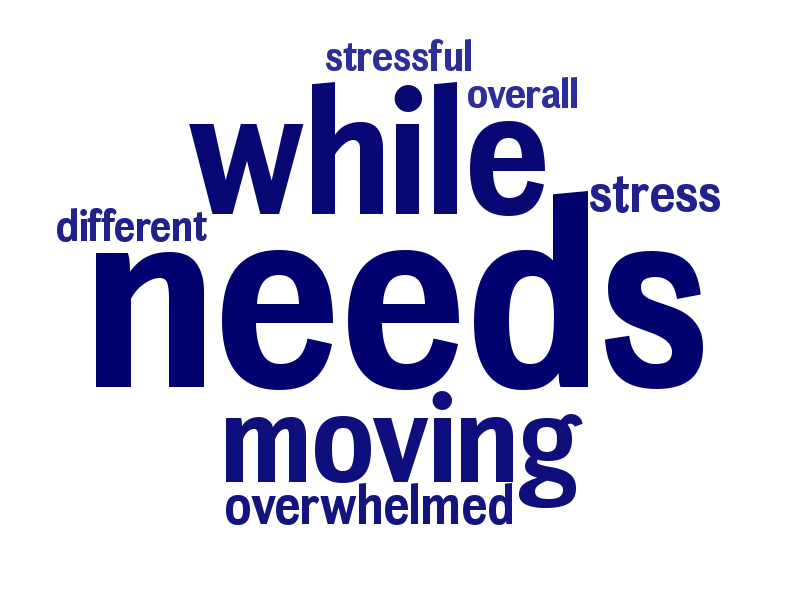

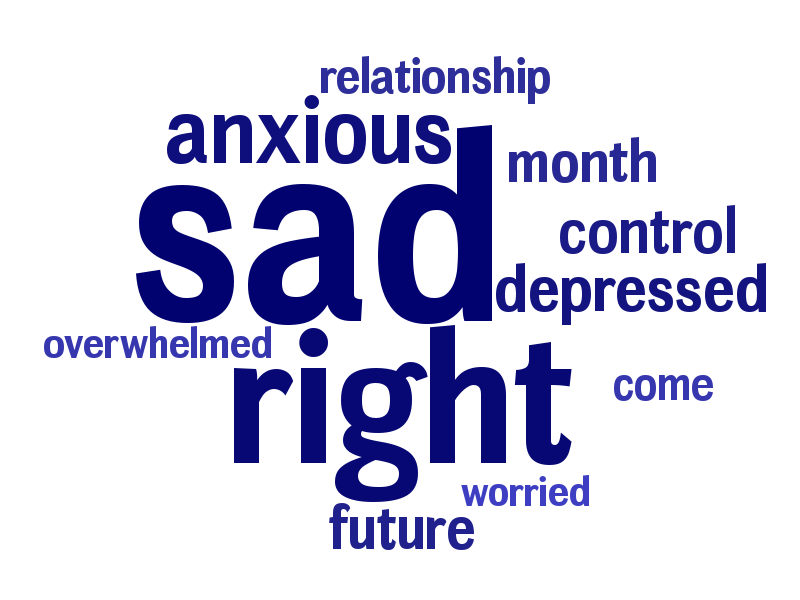

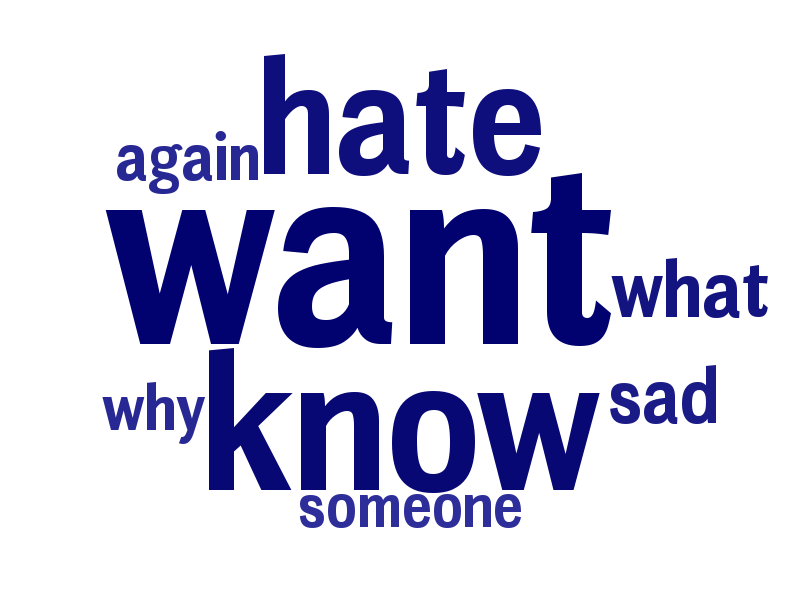
**

**Between** *r* = .27 (.26) *r* = .26 (.26) *r* = .30 (.30)

**
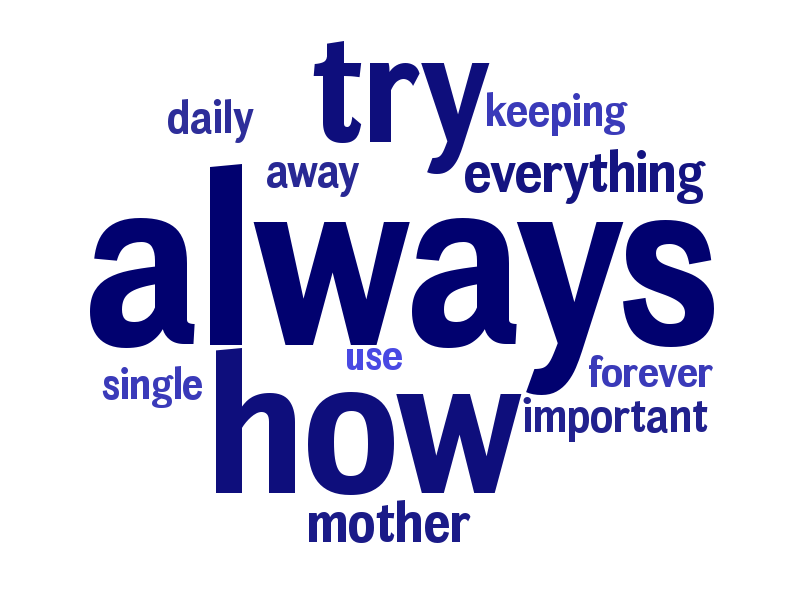

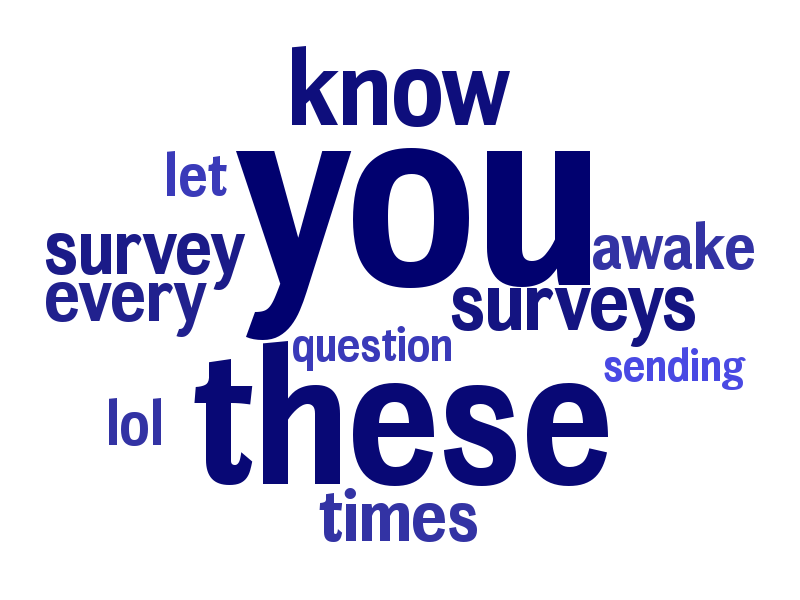
**

**Between** *r* = .27 (.27) *r* = .30 (.31)

**None of the below topics were corrected for multiple comparisons!!!**

**
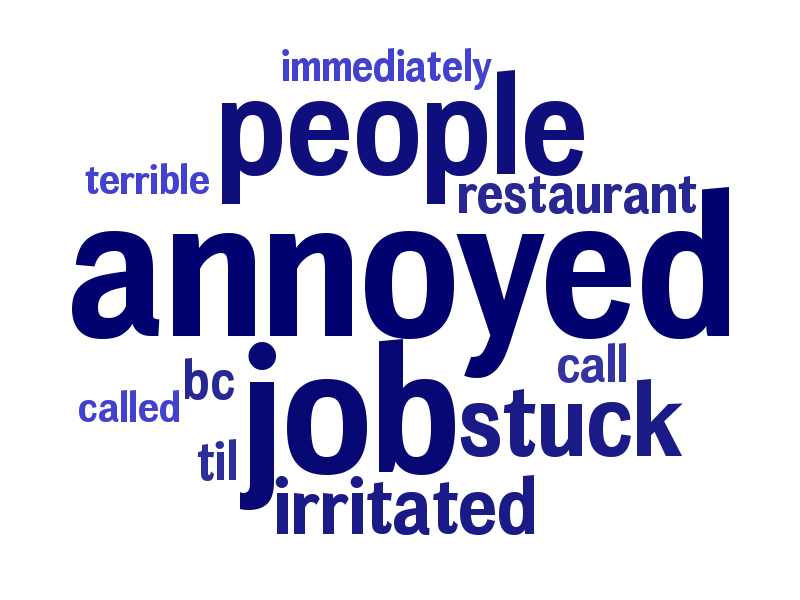

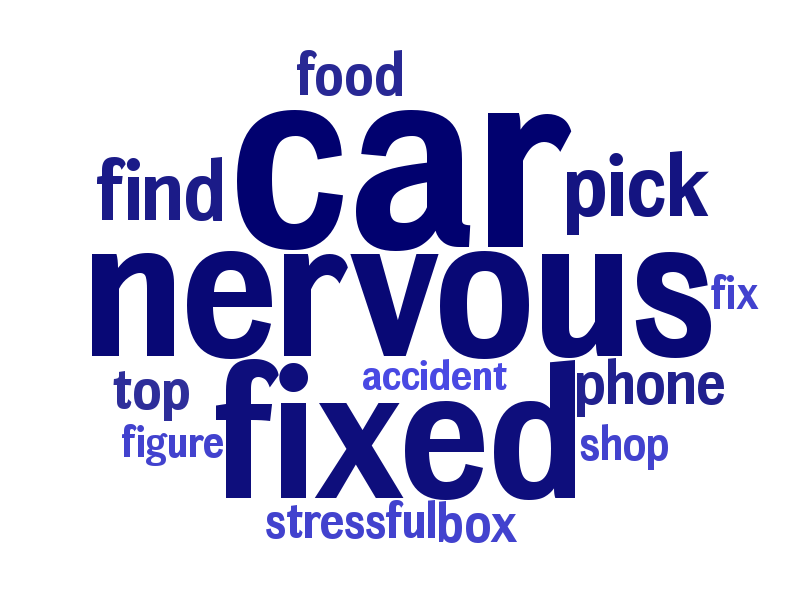

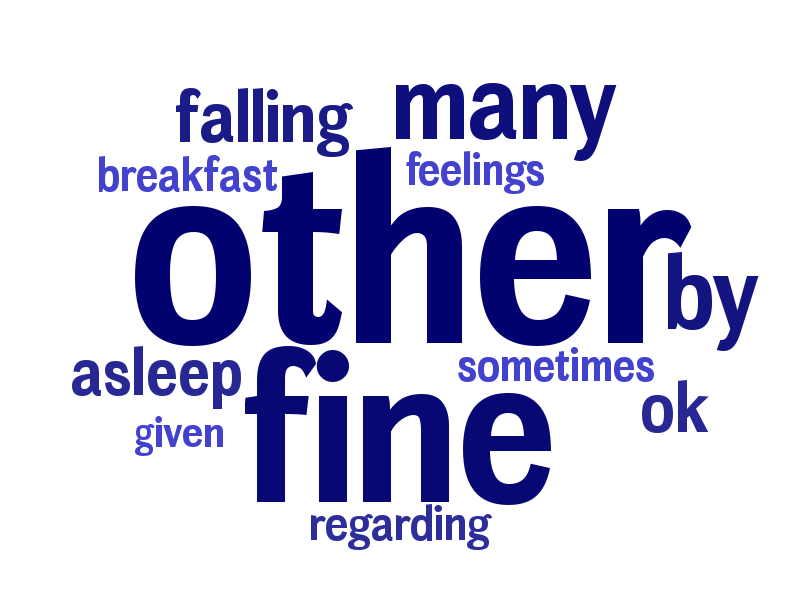

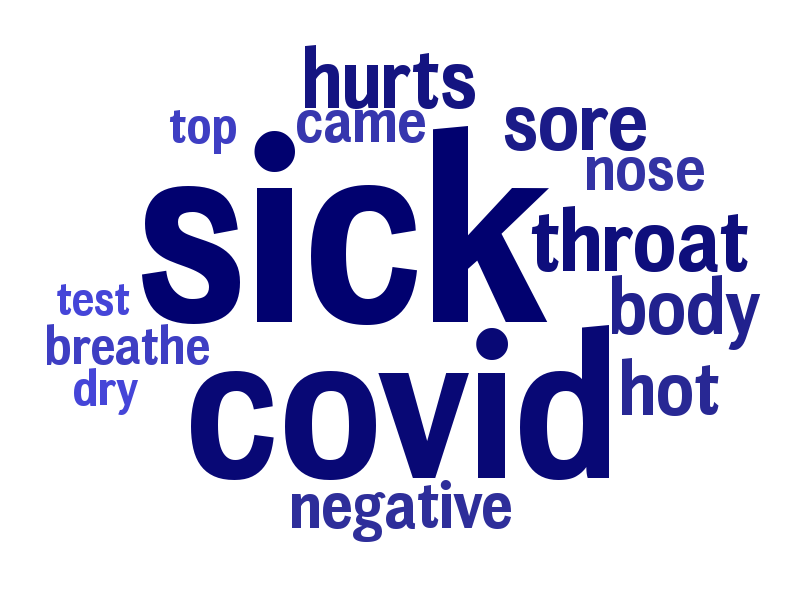

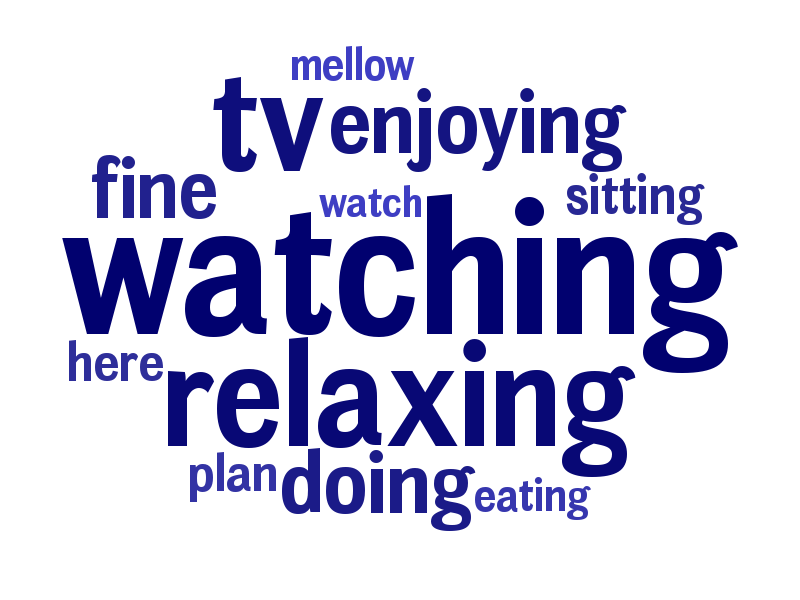
**

**W-ton** *r* = .10 *r* = .09 *r* = .07 *r* = .07 *r* = .07

**W-yes** *r* = .06

**
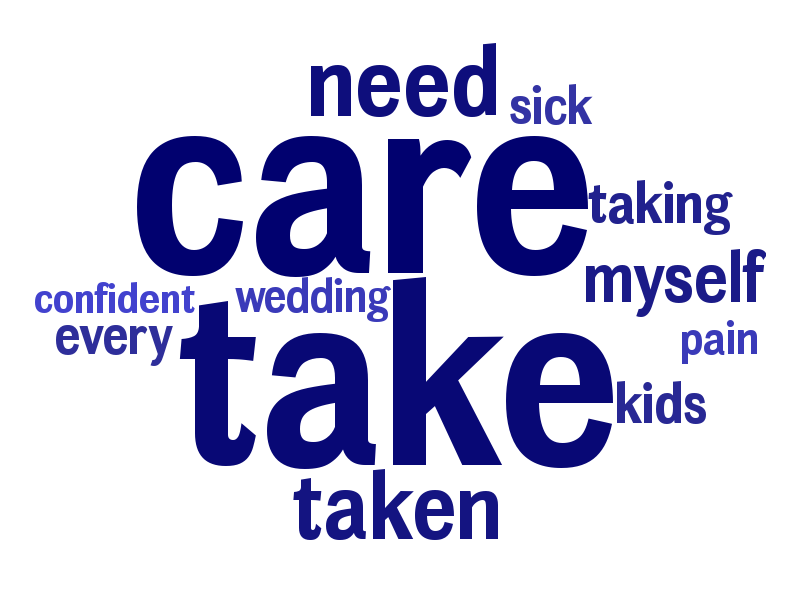

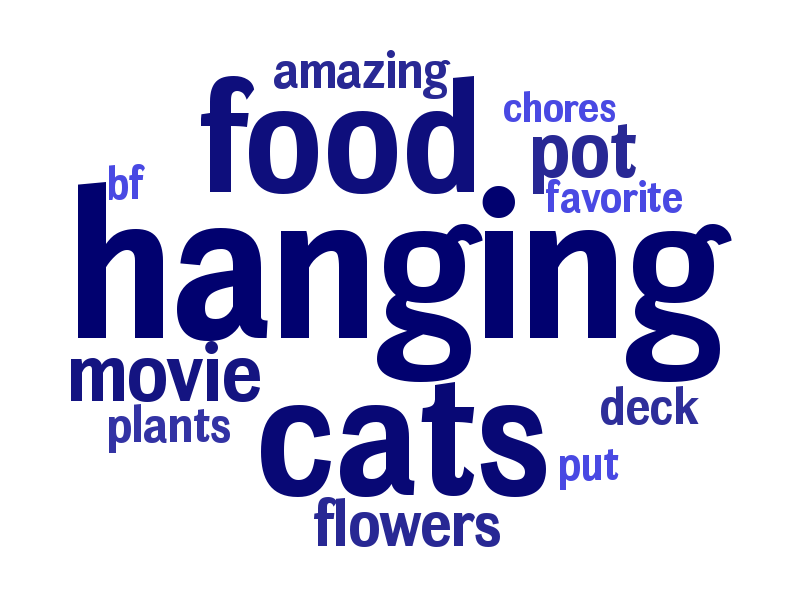

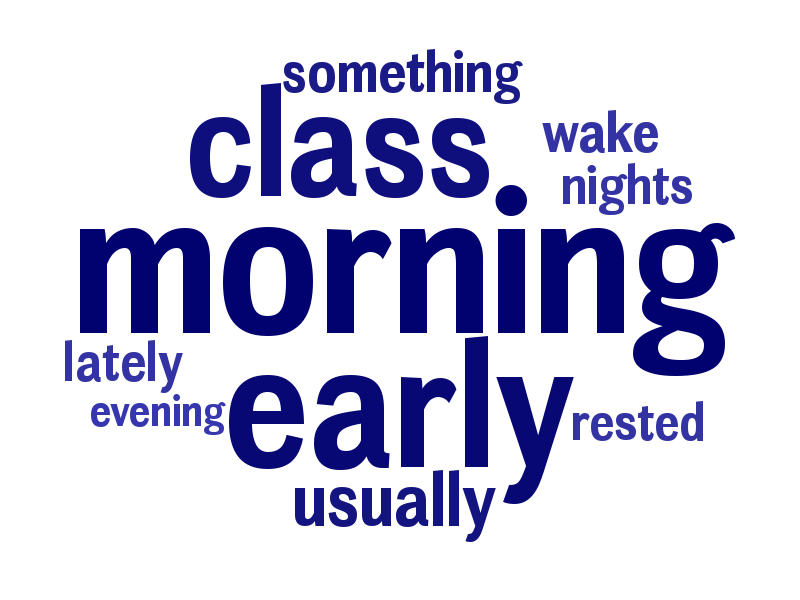

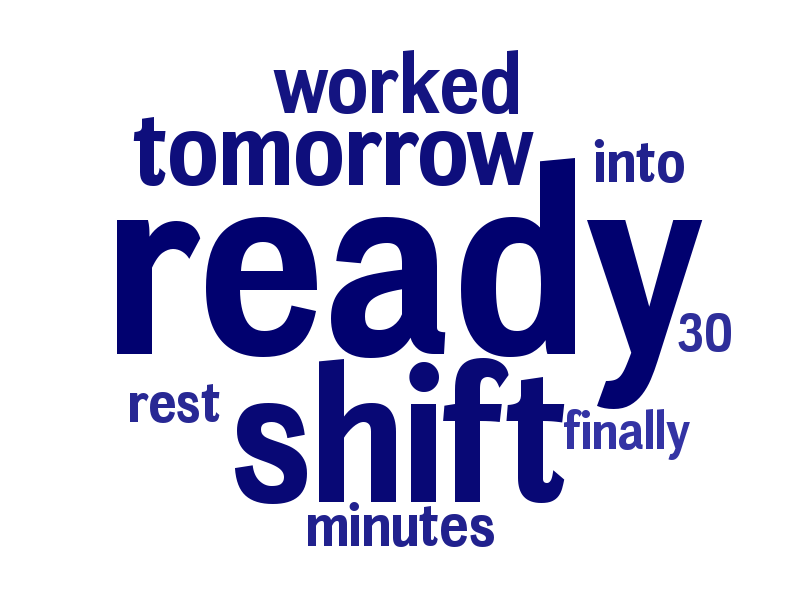
**

**W-yes** *r* = .06 *r* = .06 *r* = .06 *r* = .06

**
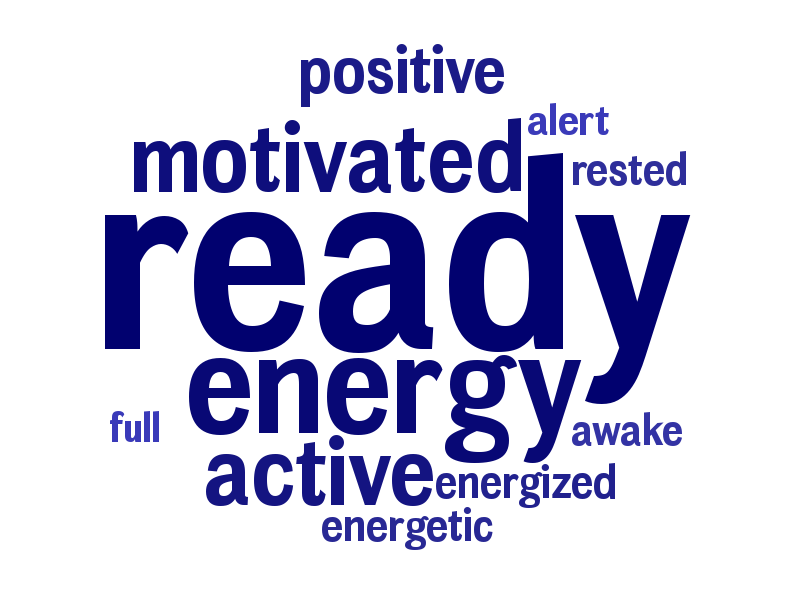

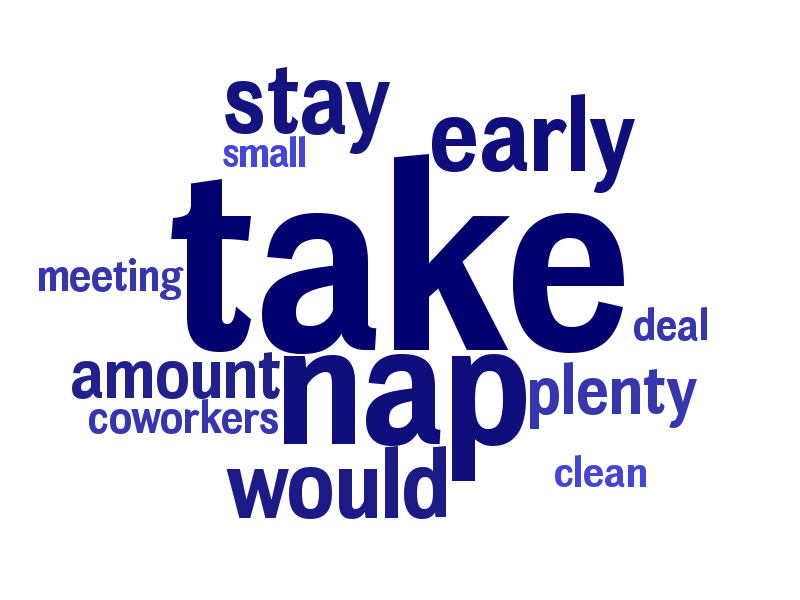

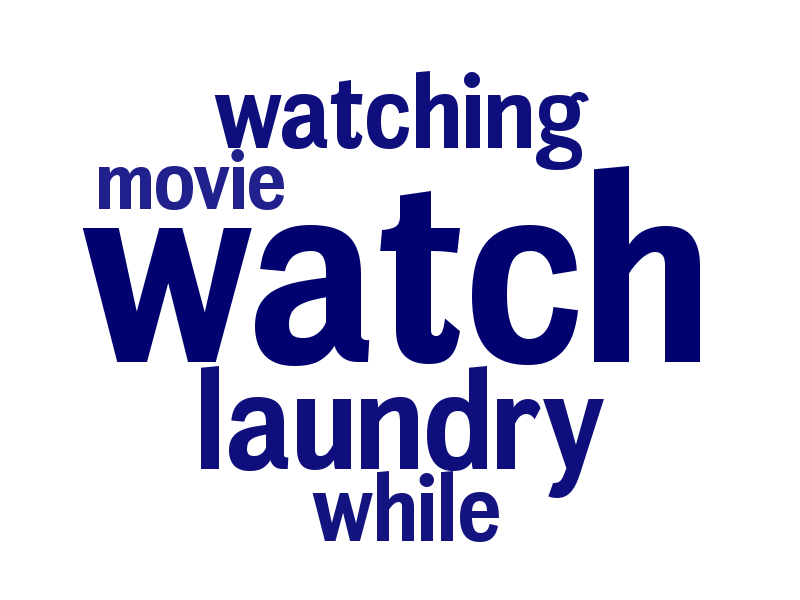

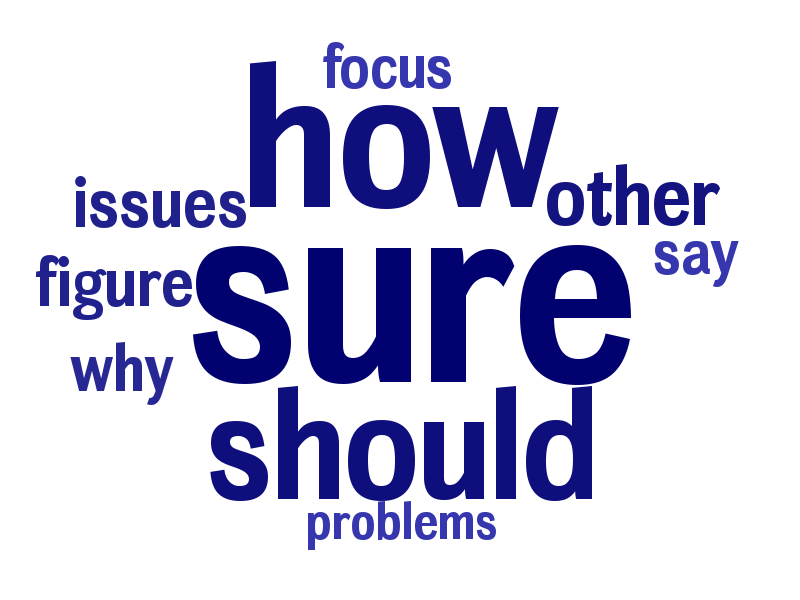
**

**W-ton** *r* = .06 *r* = .06 *r* = .06 *r* = .06

*Note:* W-yes = within person correlation to drinking yesterday, W-ton = within person correlation to drinking tonight.

**References**

1. Bates D, Mächler M, Bolker B, et al (2023). Fitting linear mixed-effects models using lme4. J Stat Softw2015;67:48. doi:10.18637/jss.v067.i01<https://www.jstatsoft.org/article/view/v067i01>
2. Bolker B, Robinson B, Menne D, Gabry J, Buerkner P, Hua C., et al (2022). Tidying Methods for Mixed Models. <https://cran.r-project.org/web/packages/broom.mixed/index.html>
3. Lüdecke, Patil, Ben-Shachar, Wiernik, Bacher, Thériault, & Makowski (2022). easystats: Framework for Easy Statistical Modeling, Visualization, and Reporting. CRAN. Available from <https://easystats.github.io/easystats/>
4. Kuznetsova, A., Brockhoff, P. B. & Christensen, R. H. B. lmerTest Package: tests in linear mixed effects models. J. Stat. Softw. 82, 1–26 (2017).
