# Supplementary material for: Language-based EMA assessments help understand problematic alcohol consumption
Source: PLoS One. 2024 Mar 6;19(3):e0298300. doi: 10.1371/journal.pone.0298300 (PMC10917301; doi:10.1371/journal.pone.0298300)
Supplement: Attachment — Submitted filename: Response to Reviewers - Language-based EMA assessments..docx [file pone.0298300.s002.docx]

**Rebuttal letter**

Dear editor and reviewers,

Thank you for your valuable feedback. We have now revised the manuscript based on your suggestions and comments. We believe that the comments we have addressed from reviewer 2 and, in particular, reviewer 1 have substantially improved the manuscript’s clarity. We have clarified that our initial results were multilevel model fixed effects implemented stage-wise through within mean-centring and that we used the Pearson correlations for greater interpretability. We have done joint mode multilevel models, and the standardised beta coefficients of these were identical to the Pearson correlations. We have also added the random effects and more mixed models with multiple predictors in the supplemental material as a result of these extra analyses. Further, when re-running the analyses, we discovered that we used the 3-item AUDIT-C and not the 10-item AUDIT in the multiple regression predicting subjective well-being (table 3). Adding the 10-item AUDIT made its std beta coefficient bump from .27 to .33. However, the overall message of this analysis did not change. The non-language data, including open code for the non-language analyses, are now available at [Open Science Framework](https://osf.io/tgvh5/).

We look forward to hearing from you again.

Our comments are in green.

**Editor’s comment**

The reviewers saw merit in the study, including seeing value in the use of language-based assessment. However, a number of issues were also raised that require addressing before the manuscript is ready for publication. In particular, Reviewer 1 highlighted the significant limitations of separate within-person and between-subjects analyses of data. Multilevel modeling is more appropriate as it can take into account the nested structure of the data. I would also suggest the authors consider PLOS ONE's data availability requirements in their resubmission.

**Reviewers' comments:**

Reviewer's Responses to Questions

**Comments to the Author**

1. Is the manuscript technically sound, and do the data support the conclusions?

The manuscript must describe a technically sound piece of scientific research with data that supports the conclusions. Experiments must have been conducted rigorously, with appropriate controls, replication, and sample sizes. The conclusions must be drawn appropriately based on the data presented.

Reviewer #1: Partly

Reviewer #2: Yes

We have addressed all of Reviewer 1’s comments and believe this issue is now resolved.

2. Has the statistical analysis been performed appropriately and rigorously?

Reviewer #1: No

Reviewer #2: Yes

We believe our clarifications and extra analyses have resolved these issues, see response to reviewer 1.

3. Have the authors made all data underlying the findings in their manuscript fully available?

The [PLOS Data policy](http://www.plosone.org/static/policies.action#sharing) requires authors to make all data underlying the findings described in their manuscript fully available without restriction, with rare exception (please refer to the Data Availability Statement in the manuscript PDF file). The data should be provided as part of the manuscript or its supporting information, or deposited to a public repository. For example, in addition to summary statistics, the data points behind means, medians and variance measures should be available. If there are restrictions on publicly sharing data—e.g. participant privacy or use of data from a third party—those must be specified.

Reviewer #1: No

Reviewer #2: Yes

We have now made the following data availability statement

**Data Availability**: The numerical data underlying the analyses, as well as the code for analysing these, are available at [Open Science Framework](https://osf.io/tgvh5/). Due to sensitivity, we can not share the language data publicly. The language data may be available on request.

4. Is the manuscript presented in an intelligible fashion and written in standard English?

PLOS ONE does not copyedit accepted manuscripts, so the language in submitted articles must be clear, correct, and unambiguous. Any typographical or grammatical errors should be corrected at revision, so please note any specific errors here.

Reviewer #1: Yes

Reviewer #2: Yes

Thank you.

**Reviewer 1.** The current paper aimed to determine how affective language reported by restaurant workers is associated with problematic and with solitary alcohol consumption. It also aimed to compare the accuracy of subjective wellbeing language measure to ratings scale measure in predicting drinking behaviour. The authors used ecological momentary assessment method to collect the data. Results showed that language-based assessments were better predictors of drinking compared to scale assessments. The study found that problematic drinking was related to reporting alcohol-related language and solitary consumption. The study also showed that on solitary drinking days, individuals reported lower subjective wellbeing, whereas social consumption days were associated with higher subjective wellbeing.

Thank you

The premise of this paper is interesting and unique. However, the structure of the paper needs to be improved, as at the moment it is lacking clarity. I also believe that additional statistical analysis will make the paper stronger.

**Abstract:**

- P.2. “Report their alcohol behavior the past 24 hours”. “In” is missing after “behavior”.

We have fixed this.

- P. 2. Conclusion: “Being alone moderated this relationship”. There was no moderation analysis conducted in this study, therefore the statement is incorrect.

We have changed “moderated” to “explained.

**Introduction:**

- The argument made in the introduction – that researchers can learn more about problematic alcohol consumption – is original and interesting. However, introduction needs to include more theory to place the argument in context. The two components of SBW are just briefly mentioned, making it harder for the reader to follow. The rationale behind using SBW, rather than separately looking at affect valence and arousal, is also not explained.

Thank you for pointing this out. We have extended the opening paragraph to add more context. The addition is in **bold**.

“Recent meta-analyses show that individuals, including those in non-clinical populations, in fact, drink more on the days they feel more positive emotions, and not when they feel more negative emotions [11,12]. **However, meta-analyses of studies of laboratory inductions of negative affect (as against controls) demonstrate small-to-medium effects on increasing alcohol use [13,14] and craving [13]. In addition to affective valence contributing to the probability of increased alcohol intake, emotion regulation strategy, and even more so, behavioural aspects of negative emotion regulation ability (e.g., impulsivity), appear to have a key role in the initiation of substance use, including alcohol across both positive and negative emotions [15].** Thus, alcohol consumption is a complex behaviour, and although it mostly relates negatively to physical health, non-problematic drinking can have a positive relationship to mental health and SWB through its strong social component [4].”

We also added a paragraph introducing SWB more to the reader, which we think will clarify.

“SWB is an important end goal for most people according to several researchers [18-20], philosophers [21], and individuals [22,23] and compose two dimensions. The affective, momentary component involves the day-to-day emotions that individuals experience [24,25], and the cognitive, evaluative component involves the overall evaluation of one’s life and is often operationalised as life satisfaction [26] or harmony in life [27]. Research on the relationship between alcohol and SWB often involves either studying how alcohol relates to daily fluctuations in affect [11,12] or to the cognitive, evaluative component of SWB [28:31]. Understanding both components, together and individually, in relation to alcohol consumption may help to understand when and how alcohol consumption can be problematic. Since SWB is highly valued among most people [22,23] and positively relates to physical health [18], it may be used to understand problematic alcohol use.”

- Rationale for using restaurant workers is not presented.

In the second paragraph of the intro, we wrote,

“We use ecological momentary assessment (EMA) with restaurant workers, a large group at increased risk for problematic alcohol use…”

To clarify this, we changed to

“We use ecological momentary assessment (EMA) with restaurant workers, a large population with higher than usual problematic alcohol drinking [16] with a particularly risky work environment involving high job stress, low job autonomy, shift-work and a drinking culture in the workplace [1,17]…”

We hope this clarifies the issue.

- Some points made by authors need to be placed in context. For example, “Most drinking occurs socially but 27% of adolescents and 40% of young adults report they have been drinking alone the past 12 months” (p. 4). What population are your referring to here? Similarly, it is unclear what is meant by “Harmony of Life” on p. 4.

In the first sentence, we have added

“, in a 4000+ US nationally representative sample,” to show what population we refer to.

In the second sentence, we have added an explanation of Harmony in Life being an approach for evaluative well-being. The sentence now reads:

“the cognitive, evaluative component [of SWB] involves the overall evaluation of one’s life and is often operationalised as life satisfaction [26] or harmony in life [27]. ”

- Some claims are not referenced, for example:

o “restaurant workers, a group at increased risk for problematic alcohol use” (p. 3).

o “non-problematic drinking can have a positive relationship to mental health and SWB through its strong social component” (p. 3).

o “individuals drink because of different reasons and because of the hump- shaped relationship drinking has to Life Satisfaction” (p. 5).

We have included references to all of the statements.

- Several key papers on the topic are missing in the introduction. I encourage the authors to consider the following publications in their literature review:

Bresin, K., Mekawi, Y., & Verona, E. (2018). The effect of laboratory manipulations of negative affect on alcohol craving and use: A meta-analysis. Psychology of Addictive Behaviors, 32(6), 617.

Tovmasyan, A., Monk, R. L., & Heim, D. (2022). Towards an affect intensity regulation hypothesis: Systematic review and meta-analyses of the relationship between affective states and alcohol consumption. PloS one, 17(1), e0262670.

Tovmasyan, A., Monk, R. L., Sawicka, I., & Heim, D. (2022). Positive but not negative affect is associated with increased daily drinking likelihood in non-clinical populations: systematic review and meta-analyses. The American Journal of Drug and Alcohol Abuse, 48(4), 382-396.

Weiss, N. H., Kiefer, R., Goncharenko, S., Raudales, A. M., Forkus, S. R., Schick, M. R., & Contractor, A. A. (2022). Emotion regulation and substance use: a meta-analysis. Drug and Alcohol Dependence, 230, 109131.

Thank you for highlighting this. We have included all the references in the manuscript.

- Please use past tense when describing the study.

Thanks for addressing this. We have edited the language when describing the study.

- “More than half of the 38 authors expected drinking to be higher on days with more negative affect”. “Authors” needs to be replaced by “studies”. (p. 3).

The claim is based on an internal survey the authors of the meta-analysis did during pre-registration (see Dora et al., 2023, *p*.14).

We have clarified the sentence to reflect this:

“At the pre-registration stage of the meta-analysis, more than half of the 38 authors of the meta-analysis reported in a survey that they expected drinking to be higher on days with more negative affect.”

We hope this is clear now.

- Please avoid using causal language, for example in “high consumption of alcohol can lead to an Alcohol Use Disorder (AUD)”, “can lead” needs to be replaced with “may be associated with” (p. 3). Similarly, “A recently published 17-year longitudinal study confirmed these effects” (p. 4). “Confirmed” needs to be replaced with “supported” or similar.

We have edited this.

- “Individuals who drink more alcohol and drink more alone in general” (p. 4) needs to be replaced with “…who drink more alone and who drink more in general”.

We have edited this.

- To guide the reader, clear aims, research questions, and hypotheses need to be identified right before the methods section.

We have added a paragraph to address this issue:

“In sum, the study aims involved the following:

1) To answer the research question, “Does language-based affective SWB predict drinking behaviour more accurately than rated SWB?”. We hypothesised that the language-based SWB would be more accurate.

2) We asked, “What is more negatively related to SWB, drinking or drinking alone?”, where we hypothesised that drinking alone would be more negatively related to SWB.

3) We aimed to answer the research question, “What specific language relates to drinking behaviour?”.“

Methods:

- Please use past tense throughout.

We have edited this.

- Data availability statement is missing.

We have added the following statement:

**Data Availability**: The numerical data underlying the analyses, as well as the code for analyzing these are available at [Open Science Framework](https://osf.io/tgvh5/). Due to sensitivity, we can not share the language data publicly. The language data may be available on request.

- “Procedure” heading is missing. Procedure section is lacking necessary details. For example, “participants were notified one to three times a day” (p. 5) –what was it dependent on? “…the text data participants provided at times included sensitive personal data” (p. 5). How was it dealt with? Further, procedure section needs to be structured better, as at the moment it is one big paragraph than needs to be split into two or three.

Thank you for pointing out the ambiguities. We have added a procedure heading, split the paragraph in three, and added:

“Participants were randomly assigned (50/50) to report EMA once or thrice a day during the two weeks after the baseline/wave survey”

and

“The data was kept in remote machines with restricted access and only some of the researchers from the larger project had access to the sensitive language data.”

- “When analyzing Drinking Tonight, a total of 10171 EMA days remained” (p. 5). This is confusing, as there is no description of “drinking tonight” variable up until this point. This later comes up in “statistical analysis section”, but a brief description of what it is needs to be presented here.

Thank you for pointing this out. Before this, we have added:
“We analyzed both Drinking Yesterday and Drinking Tonight. Since the original drinking question asked participants how much they drank in the past 24 hours, we used the language of day *n* with the reported drinks of day *n*+1 to analyze Drinking Tonight.”

- Materials section is not clear. Baseline section should precede EMA section. Each measure, including EMA alcohol measures, need to have its own subheading, there needs to be a description of what exactly does it measure, where was it adapted from (or whether it was developed for this study, and, if so, what was the process), and information on internal consistency, reliability, and validity of the measures.

Thank you for pointing this out. We have restructured the materials section according to your suggestions.

- “Two-thirds of the time, participants were asked to rate their stress and burnout” (p.6). Why two-thirds of the time?

Thank you for pointing this out. We have added a footnote describing the reason:

“Since participants either described their feelings in 3 to 5 words or 2 to 3 sentences and were asked to complete EMA’s 3 times a day, stress and burnout-related questions were only asked at the start and end of their day in order to minimize the redundancy of the questions.”

- “These scales were assessed both at baseline and in each wave survey” (p. 6). This is confusing. How many times did participants have to do baseline survey? Please describe this in the procedure section.

Thank you for pointing this out. We have added in the procedure section:

“Most participants partook only in the first wave (66%), a fourth partook in two or all three waves (26%), and some participants only partook in the second or final third wave (8%).”

This is preceded by

“At the beginning of each wave, new participants filled in an initial baseline survey, and recurrent participants took a wave survey with the same questions as the baseline except for questions about stable traits, such as sex.”

We hope that this clarifies.

- Part of the “statistical analysis” section belongs in “materials” section.

We have moved a substantial part of the analysis section that discusses the construction of the variables, including the SWB composite and the drinking variables, to the materials section.

- Is calculating SWB in such way an established procedure, or was it developer specifically for this study?

We have added the following to clarify the issue

“Although different composite scores of SWB are common [65,66], this is the first time this specific composite score of SWB has been used.”

- Language-based assessment method will be unfamiliar to some readers, therefore, more details on it are required. For example, “We used the large language model Roberta Base [53], layers 11 and 12” (p. 8) – what are layers 11 and 12? “We created 200 topics based on the co-occurrences of all the language data in our study based on 1-grams” (p. 8) – what does 1-grams refer to?

Thank you for stating this. We have added a footnote about layers stating,

“Roberta Base represents each token (i.e., word) with 12 layers. Each layer represents a slightly different semantic value depending on the context, and layers 11 and 12 have shown the best performance in syntactic and semantic structure [87], relevant to human-level tasks.”

We also added an example (new text in **bold**) of n-grams when we define it

“Latent Dirichlet Allocation [53] is an algorithm that computes how n-grams (i.e., n consecutive words; **e.g., 1-grams are single words and 2-grams are word pairs**) co-occur…”

- The software used for the analyses need to be placed at the beginning of the “analytic strategy” section.

We have replaced this.

Results:

- I appreciate conducting separate analysis of within- and between-person data. However, I think it would make more sense to conduct multilevel modeling analysis of the available data, as it would be more nuanced and informative.

Thank you for pointing this out. We see that it was not clear that the within-centred correlations represented in Tables *1b-c* were, in fact, multilevel models (fixed effects) implemented stage-wise such that a Pearson correlation could be used for greater interpretability. We have clarified in the statistical analysis,

“These within-centred correlations were multilevel model fixed effects implemented stage-wise, equivalent to the standardised beta coefficients from multilevel models implemented with joint mode (the stage-wise approach we used is accurate as long as the number of participants is at least 30 [82]). We included the joint mode within-centred multilevel models in the supplemental material (S4 Table), two models predicting within-centred SWB from within-centred day-to-day-drinking and grand mean centred AUDIT (S5 Table), and four grand-mean centred multilevel models predicting SWB from day-to-day drinking and AUDIT scores (S6-7 Tables)“,

and explain our choice of the Pearson correlations,

“We reported Pearson correlations exclusively in this paper for comparison reasons, although standardised beta coefficients would have been equally suitable for the within-person correlations, and logistic regression would have suited better in some cases, such as classifying drinking alone days.”

We hope that this clarifies.

- “The aggregated language assessments of SWB further predicted the AUDIT scores more accurately than SWB ratings did” (p. 9). The use of word “predict” is inappropriate, as this is correlational analysis.

Thank you for raising the inambiguity. The Pearson correlations are the evaluation of the accuracy of the predictive models. We have clarified this in the method section, where we added the sentence,

“Thus, we used Pearson correlation to evaluate the accuracies of the predictive models.“

- “…a strong convergence with rated SWB both between (r = .69) and within individuals (r = .57, not shown)” (p. 9). Why did you decide to not demonstrate .57 correlation in the table?

We understand that this might appear odd. We added a footnote explaining why we excluded this from the table.

“We report the within-person relationship between rated and language-based SWB in running text to demonstrate their convergence. We do not show it in Table 1d because it would only make sense to show this in Table 1d under the column “Drinking yesterday” since predicting tomorrow’s rated SWB from today’s language-based SWB is not a part of this paper.”

- “Table 1. Correlations of drinking and between and within individuals” (p. 9). “And” before “between” should be removed.

We have edited this.

- Table 1c – I was surprised to see “arousal” as a variable, as it did not come up in the analytic strategy.

After reading your comment and looking through the manuscript, we see that arousal does not add much to the narrative, so we excluded these analyses.

- Table 1d – “between” column should be renamed to “Between-person analysis of drinking” or similar.

We have edited this.

- Table 2 (p. 10) – SE is missing for most variables.

We have added this.

- P. 10 – full stop is missing when reporting .29 and .10 correlations.

We have edited this.

- “r = NS vs .09” (p. 10) – what is NS referring to?

NS = non-significant. We have spelt this out.

- Please report all correlations in the tables, even if they are not significant.

We have edited this.

Discussion:

- Discussion does not address existing theory to show how current findings could take it further.

Thank you for pointing this out. We have now added a paragraph at the end of the discussion,

“Although the negative consequences of alcohol on physical health are well documented [1-3], more and more evidence suggests that the relationship between psychological health (e.g., SWB) and alcohol consumption is more nuanced, where individuals are happier on drinking days [11-12] and low to moderate drinkers are often the happiest [28-30]. The language-based insights from the current study underscore that the social aspects of drinking are vital to understanding its relationship with psychological health and SWB. Although alcohol has been documented as a risk for mortality [2], a lack of social relationships has been at least a strong risk factor for mortality [90]. Taken together, the data, in combination with recent studies [11-12], points toward two clear distinctions of alcohol use for its impact on psychological health and SWB, namely, to what extent it is excessive and to what extent it is social. Both excessive drinking and drinking alone have been documented on how they decrease psychological and physical health [1-4,43-44], whereas fewer studies have pointed towards the potential positive impact low to moderate social drinking may have on psychological health and SWB.”

- I was confused by what is meant by “mere drinking”.

We meant drinking that was neither done alone nor problematic (AUDIT). We removed all instances of “mere” to avoid confusion. Thanks for addressing this.

- “AUDIT was distinct from drinking alone in our analysis. Both in their correlation (table 1a), but also in terms of the language they related to (tables 3 and 4)” (p. 13). These two sentences need to be combined into one.

We have edited this.

- “repeating this behavior over time might be what leads to problematic drinking in the long run” (p. 13) – please provide references to support this claim.

We have added a parenthesis after this claim clarifying how the reasoning aligns with previous research:

(in line with the longitudinal predictions of AUDIT from drinking alone [34,46])

Other:

- More cautious language needs to be used throughout the manuscript. E.g., “friends who are essential for subjective wellbeing” (p. 2), “the happiest people spend the least time alone” (p. 4), “This is evident in comparison to drinking, which in general is a very social behavior” (p. 14) are definite claims and references to back them up are not included.

Thank you for pointing this out.

**Example 1** refers to the abstract, and PLOS guidelines state, “[Do not include citations in abstracts.](https://journals.plos.org/plosone/s/submission-guidelines)”

However, we clarified that some of our references include friendships in the introduction (change in **bold**).

“Yet, alcohol consumption often occurs in social settings [4] and social connectivity**, including friendships,** serves as one of the strongest predictors of subjective well-being (SWB; [5–7])”

**Example 2**: we have clarified the referencing here, and it now reads

“Individuals high in SWB spend less time alone [6], and in one study sample, there was not one exception among the top decile of having at least one close relationship [6]”

**Example 3** refers to the conclusion of our study and we have clarified that the statement refers to our results (change in **bold**).

“This is evident in comparison to drinking, which our data **show is generally** a very social behaviour.”

**Reviewer 2.** This is an interesting article providing a novel methodology to yield insights into the effect of alcohol consumption on subjective well-being. The analysis appears to have been performed rigorously and the results are presented clearly. The paper could benefit from some minor revisions to the language to make the text more intelligible, for example:

The following sentence on page 4 is not easy to understand, particularly the end of the sentence. "The happiest people spend the least time alone and in some study samples not shown even one exception of having at least one close relationship [6]."

We edited the sentence to

“Individuals high in SWB spend less time alone [6]: in one study sample, everyone in the top decile had at least one close relationship and in one study sample [6]”

We hope this clarifies the sentence

In the following sentence and throughout the paper, 'all responses' should be changed to 'total responses'. "And second, by analyzing the between-person variation of the aggregated language from each person’s all EMA responses we will be able to have a noise-reduced assessment of the affective SWB language that relates to individuals who drink more alcohol and drink more alone in general."

We have edited this.

There may be a better way to phrase the following sentence, as it stands it is quite difficult to interpret, relating to the use of the phrase 'drinking tonight': "We also analyzed Drinking Tonight, then we transformed the data such that the emotions reported today predict the drinking tonight that was reported tomorrow." (page 6)

We have clarified the sentence, and it now reads.

“We also analysed Drinking Tonight. To do this, we transformed the data such that the emotions reported today, day n, predict the drinking tonight that was reported tomorrow, day n+1 (since the drinking question asked about drinking the past 24 hours). “

Although there is a general ethics statement response for the paper guidelines, it might be good to describe in a bit more detail the ethics of recruiting from social media and how this took place.

We have added the following statement

**“Ethical statement.**

The study is part of a larger NIAAA-funded study with the working title, “Data Science for Unhealthy Drinking (DS4UD),” H Andrew Schwartz, PI. The study received its ethical approval from the University of Pennsylvania Institutional Review Board. NIAAA did not play a role in the study design. All participants provided digital informed consent to participate in the study. In the consent information, they were provided information specific to the study design and the details of their commitment, along with contact information for study personnel. Additionally, participants were told about their right to withdraw from the study at any given moment and told that the data would only be analysed at the group level and no sensitive data would be made public.”

The paper could do with an overall proofread – for example, 'solitary drinkers' may be better than ‘alone drinkers’.

We have changed all instances of alone drinkers to solitary drinkers, and five of the English native-speaking authors have done a proofreading checking for grammatical and semantic errors.
